# Metatranscriptomic analysis of a high-sulfide aquatic spring reveals insights into sulfur cycling and unexpected aerobic metabolism

**DOI:** 10.7717/peerj.1259

**Published:** 2015-09-22

**Authors:** Anne M. Spain, Mostafa S. Elshahed, Fares Z. Najar, Lee R. Krumholz

**Affiliations:** 1 Department of Biological Sciences, Ferris State University, Big Rapids, MI, United States; 2 Department of Microbiology and Plant Biology and the Institute for Energy and the Environment, University of Oklahoma, Norman, OK, United States; 3 Department of Microbiology and Molecular Genetics, Oklahoma State University, Stillwater, OK, United States; 4 Department of Chemistry and Biochemistry and the Advanced Center for Genome Technology, University of Oklahoma, Norman, OK, United States

**Keywords:** Metatranscriptomics, Biodiversity, Microbial mats, Sulfur cycle, Sulfur spring, Oxygenic photosynthesis, Sulfide oxidation, Methane oxidation, Actinobacteria

## Abstract

Zodletone spring is a sulfide-rich spring in southwestern Oklahoma characterized by shallow, microoxic, light-exposed spring water overlaying anoxic sediments. Previously, culture-independent 16S rRNA gene based diversity surveys have revealed that Zodletone spring source sediments harbor a highly diverse microbial community, with multiple lineages putatively involved in various sulfur-cycling processes. Here, we conducted a metatranscriptomic survey of microbial populations in Zodletone spring source sediments to characterize the relative prevalence and importance of putative phototrophic, chemolithotrophic, and heterotrophic microorganisms in the sulfur cycle, the identity of lineages actively involved in various sulfur cycling processes, and the interaction between sulfur cycling and other geochemical processes at the spring source. Sediment samples at the spring’s source were taken at three different times within a 24-h period for geochemical analyses and RNA sequencing. In depth mining of datasets for sulfur cycling transcripts revealed major sulfur cycling pathways and taxa involved, including an unexpected potential role of Actinobacteria in sulfide oxidation and thiosulfate transformation. Surprisingly, transcripts coding for the cyanobacterial Photosystem II D1 protein, methane monooxygenase, and terminal cytochrome oxidases were encountered, indicating that genes for oxygen production and aerobic modes of metabolism are actively being transcribed, despite below-detectable levels (<1 µM) of oxygen in source sediment. Results highlight transcripts involved in sulfur, methane, and oxygen cycles, propose that oxygenic photosynthesis could support aerobic methane and sulfide oxidation in anoxic sediments exposed to sunlight, and provide a viewpoint of microbial metabolic lifestyles under conditions similar to those seen during late Archaean and Proterozoic eons.

## Introduction

The combination of molecular techniques and high-throughput sequencing in microbial ecology has revolutionized the ability to probe questions regarding microbial community structure and function (Handelsman, 2004). Metagenomic and metatranscriptomic approaches have provided valuable insights into the roles of uncultivated lineages within a microbial community (Pelletier et al., 2008; Liu et al., 2012; Kozubal et al., 2013; Sheik, Jain & Dick, 2014) as well as in delineating the relative importance and contributions of various microbial taxa to observed geochemical processes occurring in habitats characterized by complex biodiversity, such as soil (Urich et al., 2008; Shrestha et al., 2009), surface and deep ocean water (Poretsky et al., 2005; Frias-Lopez et al., 2008; Poretsky et al., 2009; Baker et al., 2013), deep ocean hydrothermal vent systems (Xie et al., 2010; Lesniewski et al., 2012), and hot springs (Liu et al., 2011; Burow et al., 2013). In complex habitats like these, small-subunit (SSU) rRNA gene-based surveys and metagenomic approaches can lead to exhaustive lists of taxa detected and speculation of the potential roles of dominant taxa in metabolic processes. As such, a metatranscriptomic approach better allows for the determination of microbial populations that are actively involved in processes of interest.

In particular, metatranscriptomics represents an ideal tool to study sulfur cycling processes and the microbial lineages involved at Zodletone spring, a sulfide and sulfur-rich spring in southwestern Oklahoma. The spring itself is a sunlight-exposed shallow aquatic habitat with highly reduced sediments, in which O_2_ is undetectable (Senko et al., 2004; Bühring et al., 2011). Because of the lack of detectable O_2_, it has also been suggested that photosynthesis by *Cyanobacteria* in spring’s sediment is anoxygenic (Bühring et al., 2011). The water overlying the sediments has low O_2_ concentrations (2–4 µM) (Bühring et al., 2011), high levels of methane from the subsurface, and high concentrations of sulfur in various forms (Donovan, Younger & Ditzell, 1988).

Sulfur cycling processes at the site have been described as anaerobic. Anoxygenic sulfide oxidation appears to be light-driven and anaerobic (Elshahed et al., 2003; Senko et al., 2004), and indeed, *Chromatiales* and *Chlorbi* (the anaerobic purple and green sulfur bacteria) lineages have been detected among SSU rRNA-based surveys (Elshahed et al., 2003; Youssef, Couger & Elshahed, 2010); however, it is unknown whether these lineages are the sole contributors to sulfide and sulfur oxidation or if other lineages, such as *Epsilonproteobacteria*, which has been detected in the spring (Elshahed et al., 2003; Youssef, Couger & Elshahed, 2010; Bühring et al., 2011), might also be involved. Similarly, sulfate reduction also appears to be a dominant process in the anoxic sediments during dark periods (Elshahed et al., 2003; Senko et al., 2004), but while many anaerobic *Deltaproteobacteria* lineages known to be involved in sulfate reduction have been detected (Elshahed et al., 2003; Youssef, Couger & Elshahed, 2010), it is unclear if other sulfur-reducing processes (e.g., sulfur reduction, thiosulfate reduction or disproportionation) also occur *in situ* and which microbial lineages are involved. Indeed, Zodletone spring sediments harbor an extremely diverse microbial community (Elshahed et al., 2003; Youssef, Couger & Elshahed, 2010), but SSU rRNA-based surveys alone cannot tell us specifically which of the taxonomic lineages detected (especially those with no or few members that have been validly described) are actively involved in any of the sulfur cycling processes, or other photo- and chemotrophic processes, occurring at the site.

Thus, a metatranscriptomic approach, especially if coupled to a detailed geochemical characterization, could be extremely useful. In this study, we used metatranscriptomics to examine the roles of various microbial lineages in processes occurring in Zodletone spring sediments throughout the course of a day, with the goal of identifying microorganisms involved in general photo- and chemotrophic processes as well as sulfur cycling processes. The data presented here highlight taxa involved in a complex sulfur cycle and also provide some unexpected findings regarding the production and utilization of oxygen in the anoxic sediments at the spring’s source.

## Materials and Methods

### Site description and geochemistry

Zodletone Spring, located in Kiowa County (Oklahoma; Coordinates: 35.002470, −98.688091), is an anoxic, barite-depositing spring with a highly diverse microbial community (Elshahed et al., 2003; Elshahed et al., 2004; Youssef, Couger & Elshahed, 2010), driven largely by sulfur cycling processes (Elshahed et al., 2003; Senko et al., 2004). The spring source, approximately 1 m^2^, feeds a small stream (∼20 m), which empties into nearby Saddle Mountain Creek (Fig. S1). Spring source water is barium- (∼0.3 mM) and sulfide-rich (8–10 mM), bubbling continuously with methane and short-chain alkanes, and has a unique chemical composition, largely due to its origin, a mixture of deep Anadarko basin brine water and shallow groundwater (Donovan, Younger & Ditzell, 1988). Spring water originates underground and has a constant year-around temperature of 22 °C (Younger, 1986; Youssef, Ashlock-Savage & Elshahed, 2012) and also contains sulfate (∼60 µM), and moderate levels of NaCl (∼0.2 M) (Senko et al., 2004).

The soft, black, biomass-rich sediments at the spring’s source are immobile and represent a stable environment. Although it was previously reported that spring water is anoxic (Senko et al., 2004), a more recent study using microsensors found that though oxygen was not detectable within and directly above (<1 mm) the source sediment, concentrations increased to 2–4 µM at 2 mm above the source sediment-water interface; therefore, source sediments and overlying waters can be classified as anoxic and micro-oxic (<5 µM), respectively (Bühring et al., 2011).

### Sampling description

Zodletone source sediment and groundwater were sampled in early November 2009 over a 24 h period to catch the diurnal fluctuations in sulfur cycling processes previously observed, i.e., that sulfide oxidation and production appear to occur during sunlit vs. dark periods, respectively (Elshahed et al., 2003; Senko et al., 2004). Conditions on the day of sampling were sunny and mild, with air temperatures recorded as follows for each time of sampling: 24.4 °C at 12:15 (daytime; peak sunlight), 22.8 °C at 17:15 (early evening; one half hour before sunset), 15.6 °C at 22:15 (night; full darkness), and 15.0 °C at 07:30 (early morning; one half hour after sunrise). At each of these time points, source sediment (approx. 5 g) was aseptically collected, preserving transcripts by immediate (within five seconds) on-site immersion of sediment in 9 mL LifeGuard™ Soil Preservation Solution (MoBio Laboratories, Inc., Carlsbad, CA). Samples were mixed well then frozen by submersion in an ethanol bath super-cooled with dry ice. Using this procedure, each sediment sample-LifeGuard mixture was frozen in under one minute. Each sample was collected from the top layer (5 cm) of sediment from the south-facing corner of the spring (Fig. S1), directly adjacent to the spring’s source of water (as identified by visible bubbling), where the sediment has a uniform appearance, characterized as soft, black, with few to no leaves present. Care was taken to ensure minimal disturbance, and subsequent samples were obtained, using the same techniques, from the same location and depth immediately adjacent to the original site sampled. All sediment samples were transported on dry ice to our laboratory and stored at −80 °C for RNA extraction.

Water from the spring’s source was also collected for various geochemical analyses. For pH and anion analysis, water from the source was collected, filtered through 0.2 um filter, and frozen immediately using the dry ice/ethanol bath. For sulfide preservation, water samples (2.5 ml) were collected and directly injected into serum tubes containing 2.5 ml anoxic 10% zinc acetate (N_2_ headspace). Water samples were transported either on dry ice (tubes for pH/anion analysis) or on ice (tubes for sulfide analysis) to our laboratory and stored at −20 °C (tubes for pH and anion analyses) or 4 °C (tubes for sulfide measurements). Water samples were collected, transported, and stored in a similar matter for pH and sulfide analyses again in August 2014.

### Analytical methods

From water samples, pH was measured under N_2_:CO_2_ (80:20) headspace using a pH electrode. Anion (chloride, nitrate, nitrite, phosphate, sulfate, sulfite, and thiosulfate) concentrations were determined by ion chromatography (Dionex, model DX500 fitted with the AS-4A column; Dionex Corporation, Sunnyvale, CA). To minimize oxidation of thiosulfate and sulfite, standards and sample dilutions into Dionex vials were prepared in an anaerobic glove bag (Coy Laboratory Products, Inc., Grass Lake, MI). Sulfide was measured by the methylene blue assay (Cline, 1969).

### RNA extraction, processing, and pyrosequencing

RNase-free materials and reagents were used in all RNA extraction and processing steps. RNA was extracted from sediment within two months of sampling (RNA PowerSoil^®^ Total RNA Isolation kit; MoBio). Remaining DNA was then digested (RNase-free DNase-I; MoBio), and an enzymatic rRNA removal step was performed (mRNA-ONLY Prokaryotic mRNA Isolation Kit; EPICENTRE Biotechnologies, Madison, WI). Samples were stored at −80 °C until a second rRNA-removal step, based on subtractive hybridization, was performed (MICROBExpress™ Bacterial mRNA enrichment kit; Ambion, Austin, TX). Enriched mRNA was amplified via *in vitro* transcription of synthesized cDNA (MessageAmp™ II-Bacteria Prokaryotic RNA Amplification Kit; Ambion). We quantified total RNA (ng/µl) before and after ribodepletion steps. As a control, RNA was also amplified from the non-ribodepleted RNA (RNA prior to rRNA depletion steps) fraction of one sample (12:15 time point) in order to examine the efficiency of rRNA removal, to examine the microbial community structure of the non-ribodepleted sample, based on analysis of SSU rRNA gene transcripts present, and to describe the effects of ribo-depletion on SSU rRNA-based microbial community structure.

Amplified RNA was assessed for quality and quantity by both denaturing gel electrophoresis and spectrophotometric analysis (NanoDrop Products, Wilmington, DE). The quality of the 17:15 was too poor for sequencing; thus, library preparation and sequencing via Roche Titanium 454 pyrosequencing was conducted from the remaining three samples (12:15, 22:15, and 07:30), using the service of a commercial provider (MOgene, LC., St. Louis, MO).

### Sequence handling and analyses

(i) *Sequence data handling*. Raw sequence data (.fna and .qual files) were uploaded to MG-RAST (v2) (Meyer et al., 2008) for data handling and functional annotation. From these total reads datasets, several data subsets were also created. Microbial SSU rRNA data subsets were created by downloading reads from MG-RAST with significant alignment (min. alignment length = 50 bp; max. E-value = 1E–30) to sequences from within the Greengenes (*Bacteria*) and SILVA SSU (*Archaea*) databases. Potential mRNA data subsets from each ribodepleted sample (12:15, 22:15, and 07:30) were created by removing all sequences with significant similarity (max. E-value = 10E-3) to bacterial, archaeal, or eukaryotic large and small subunit ribosomal RNA sequences, as identified using the BLASTN alignment tool against the nt database (Altschul et al., 1997). As well, reads from potential mRNA data subsets were assembled for more detailed analysis of sulfur and oxygen cycling genes. To assemble the raw reads, we performed three separate assemblies using Newbler, the 454 assembly software, on untrimmed reads, reads trimmed at 84, and reads trimmed at 63 cycles. The three different trimming lengths were used to reduce the number of artificial contigs produced due to poor qualities at the end of the contigs (Wiley et al., 2009). The contigs from the three separate assemblies were combined and further assembled with Phrap (Ewing & Green, 1998). Potential mRNA and assembled datasets were also uploaded to MG-RAST (v2) for analyses.

(ii) *Taxonomic classification of SSU rRNA reads*. Taxonomic assignment of each SSU rRNA read was performed using the on-line Classifier tool available from the Ribosomal Database Project (Wang et al., 2007). In order to characterize the effect of the rRNA removal steps on microbial community analysis, microbial community composition (characterized by the number of taxa detected at the phylum, class, and order levels) of the 12:15 total RNA control sample was compared to that of its (12:15 time point) corresponding ribodepleted dataset. Population distribution at each taxonomic level was also determined using relative frequencies of each taxon, and the two samples were statistically compared by a chi-square test based on an r × k contingency table.

(iii) *Taxonomic assignment of mRNA transcripts*. A BLASTX-MEGAN based analysis was used to assign NCBI taxonomic affiliations to mRNA transcripts. First, sequences from potential mRNA data subsets were examined using the BLASTX search query against the NCBI nr database; results (top 5–10 most closely related hits with a max. E-value cutoff of 10E-5) were saved and summarized in searchable tab-delimited text files. The BLASTX results produced were then used by MEGAN, v4 (Huson et al., 2011) to assign a taxonomic affiliation to each mRNA transcript. Sequences in which top BLASTX hits identified with different taxonomic lineages (i.e., sequences with similar functions but belonging to different phyla) typically remained “taxonomically unclassified” by the program, thus minimizing the chances of false classifications that might occur if only the top BLASTX hit had been used.

(iv) *Functional analyses of mRNA transcripts*. Potential mRNA data subsets were analyzed in MG-RAST, v2, for functional gene annotation and SEED subsystem determination (Meyer et al., 2008), using the following parameters: minimum alignment length, 34 bp; maximum E-value, 0.01. A number of rRNA sequences remained in these datasets; thus, the final number of potential mRNA reads for each dataset was determined by subtracting the number of sequences with significant alignments (min. alignment length = 50 bp; max. E-value = 1E–30) to SSU and large subunit (LSU) rRNA databases from the total number of sequences. Functional gene assignments from each subsystem were downloaded as sortable, searchable spreadsheets.

Additionally, individual reads and assembled contigs coding for genes putatively involved in sulfur and oxygen transformations were identified by mining all data files (MG-RAST annotation spreadsheets, MEGAN files and BLASTX summary files) for genes identified in MetaCyc pathways (Caspi et al., 2008) or by primary literature as being involved in metabolisms of interest, and subjected to further in-depth analysis.

### Nucleotide sequences accession numbers

Sequences for each of four samples were deposited to GenBank through the sequence read archive and can be retrieved from the following accession numbers: SRX025760, SRX025825, SRX025796 [corresponding to ribodepleted 22:15 (night), 07:30 (early morning), and 12:15 (afternoon) samples, respectively], and SRX331735 (corresponding to the 12:15 non-ribodepleted RNA control sample). Various datasets are publicly available in MG-RAST (v3) under the following Sample ID numbers: original RNA datasets (without rRNA sequences removed) from 22:15 (night), 07:30 (early morning), and 12:15 (afternoon) samples correspond to project IDs 4450335.3, 4450336.3, and 4450338.3, respectively; potential mRNA datasets (with rRNA sequences removed) from 22:15 (night), 07:30 (early morning), and 12:15 (afternoon) samples correspond to project IDs 4451038.3, 4451039.3, and 4451037.3; assembled mRNA datasets from 22:15 (night), 07:30 (early morning), and 12:15 (afternoon) samples correspond to project IDs 4453253.3, 4453260.3, and 4453251.3; finally, datasets from the non-ribodepleted control RNA sample (12:15) correspond to Project IDs 4451467.3 (all RNA reads) and 4451718.3 (potential mRNA dataset).

## Results

### Geochemistry of Zodletone spring water

**Figure 1:**
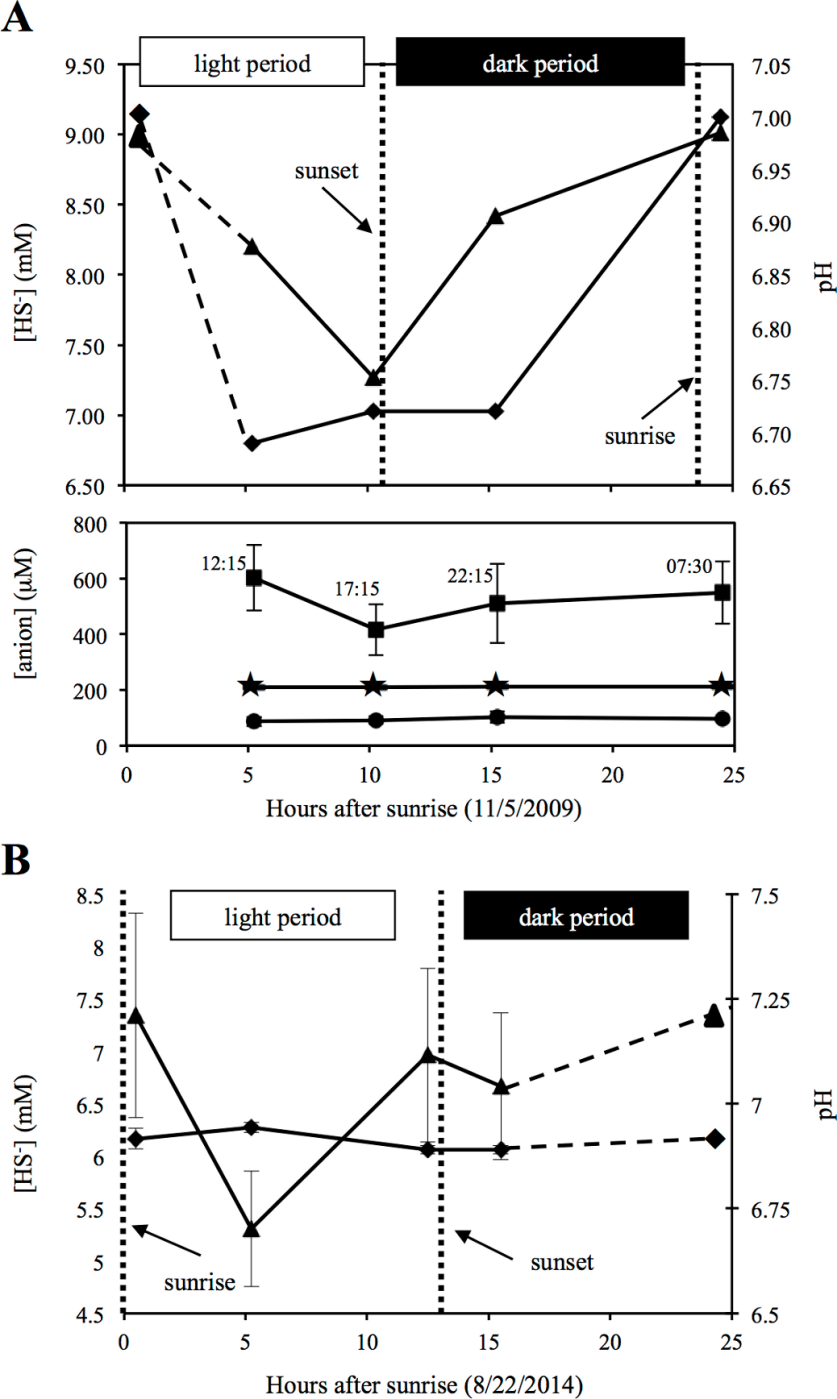
Geochemistry of Zodletone spring’s source water. Geochemistry of Zodletone source water over the time course of the experiment is shown at the top (A). Dashed lines indicate an extrapolation of the data from the 07:30 (early morning) time point. Dotted vertical lines indicate sunset and sunrise on day(s) of sampling. Above: triangles and diamonds indicate sulfide concentrations and pH values, respectively. Below: squares, stars, and circles indicate thiosulfate (S_2_O_3_^2−^), sulfite (SO_3_^2−^), and sulfate (SO_4_^2−^) concentrations, respectively. Y-error bars represent the standard deviation of duplicate measurements for sulfate and triplicate measurements for thiosulfate. Sulfite, pH, and sulfide values were based on single measurements only. Source water was sampled again in August 2014 to measure pH and sulfide in triplicate and verify patterns of sulfide loss and generation during the day and night, respectively (B).

Sulfide measurements from source water samples at the time of sampling (Nov, 2009) and again in Aug, 2014, indicated a progressive decrease in sulfide values during the day, followed by increase in sulfide levels overnight (Fig. 1). These results confirm previous findings from two previous studies that utilized either direct geochemical measurements at the spring source or laboratory incubations of spring source sediments, and demonstrated that sulfate production from sulfide is primarily a light-dependent process, whereas sulfate reduction/sulfidogenesis occurs predominately nocturnally/in dark incubations (Elshahed et al., 2003; Senko et al., 2004). The results of these studies conducted over more than a decade, along with the documentation of a similar pattern in this study (Fig. 1), demonstrate that the observed sulfur cycling dynamics in Zodletone spring source water is an ecologically relevant and temporally stable process, with sulfur oxidative processes occurring during the day and reductive processes increasing at night.

Sulfate and sulfite were detected at all time points (average concentrations = 94.5 ± 7.1 µM and 211.2 ± 1.3 µM, respectively) , with little to no difference observed among the four time points (Fig. 1). Compared to these sulfur oxyanions, higher levels of thiosulfate (avg = 520.3 ± 78.76 µM) were detected, with some fluctuations observed (Fig. 1). Chloride was also detected (avg = 186.3 ± 21.4 mM), whereas nitrate, nitrite, and phosphate were not. Anion data are consistent with previous studies, conducted at Zodletone spring in November 2002 (Senko et al., 2004) and August 2005 (Bühring et al., 2011).

### Pyrosequencing dataset information

Pyrosequencing of four samples (ribodepleted 22:15, 07:30, and 12:15 samples, and the 12:15 non-ribodepleted 12: 15 RNA sample) yielded a total of 1,122,073 reads, with average read lengths ranging between 344–427 nucleotides (Table 1). Ribodepletion steps resulted in a two- to three-fold enrichment in the percentage of potential protein-coding, or mRNA reads, which comprised 11.0–17.7% of total RNA reads in the ribodepleted datasets (Table 1). From potential mRNA subsets, a total of 30,887 reads (22.2–26.6% of total potential mRNA reads) were functionally annotated by MG-RAST (Table 1). BLASTX analysis of these datasets, however, resulted in 44.7–48.9% of potential mRNA transcripts having significant (maximum E value = 1E-5) hits against the nr database (Table 1). Finally, 10.2–13.4% of the potential mRNA transcripts were taxonomically unclassified (could not be placed into any domain or phylum with certainty) (Table 1).

**Table 1:** Total pyrosequencing dataset statistics, including statistics for potential mRNA transcripts data subsets.

	Full dataset stats		Statistics for subsets of potential mRNA transcripts			
Sample/time of day	Total no. of reads	Avg. read length (bp)	No. of potential mRNA reads (% of total)	No. of potential mRNA reads with no significant BLASTX alignments (%)	No. of potential mRNA reads functionally annotated into MG-RAST subsystems (%)	% potential mRNA reads taxonomically unclassified^a^
22:15	297,646	427	34,835 (11.7%)	15,935 (45.7%)	7,730 (22.2%)	10.2%
07:30	301,933	412	53,381 (17.7%)	26,079 (48.9%)	14,212 (26.6%)	10.4%
12:15	324,282	402	35,643 (11.0%)	15,938 (44.7%)	8,945 (25.1%)	13.4%
12:15 (total RNA control)	198,212	344	10,035 (5.1%)	–	–	–

**Notes.**

a Unassigned at the phylum-level and includes the sum of all potential mRNA reads assigned by MEGAN 4.0 as Unassigned Bacteria, Unassigned Archaea, and Unassigned Cellular Organisms.

### Taxonomic classification of SSU rRNA reads and the effect of rRNA depletion on microbial community structure

Microbial community structure from each SSU rRNA data subset was evaluated by phylogenetic analysis of SSU rRNA reads. Results from our non-ribodepleted sample showed a remarkably similar microbial community structure to what has been described in the past (Elshahed et al., 2003; Elshahed et al., 2004; Youssef, Couger & Elshahed, 2010), with dominant phyla including *Proteobacteria*, *Bacteroidetes*, *Firmicutes*, *Actinobacteria*, *Spirochaetes*, *Euryarchaeota*, and *Chloroflexi*. Candidate divisions (e.g., BRC1, OP11, OD1, WS3) and several “rare phyla” (i.e., taxa that comprise <0.1% of total microbial community), defined in a previous study from Zodletone (Youssef, Couger & Elshahed, 2010), were also represented in SSU rRNA subsets (Fig. S2 and Table S2). For example, although the phylum *Planctomycetes* had been previously determined to comprise 0.01–0.1% of the microbial community in Zodletone source sediment (Youssef, Couger & Elshahed, 2010), *Planctomycetes*- affiliated SSU rRNA sequences comprised 0.563% of the total SSU rRNA sequences from our non-ribodepleted SSU rRNA dataset and 1.45–2.15% of sequences from ribodepleted datasets SSU rRNA (Table S2).

The removal of ribosomal RNAs did not appear to reduce the number of taxa detected at phylum, class, or order levels (Tables S1 and S2). For example, 31 phyla were detected in both ribodepleted and non-ribodepleted RNA samples from 12:15 (Tables S1 and S2). However, though similar taxa are detected from non-ribodepleted vs. ribodepleted samples (Fig. S2), the two microbial community structures, based on rel. frequencies of each taxon, were significantly different at the phylum, class and order levels (*p* < 0.001). Thus, as the ribodepletion steps inherently increase the error associated with comparing the relative abundance of one taxonomic group to that of others within a sample, ribodepleted datasets should be limited to only the detection of active microbial taxa in a sample and not to estimate taxon abundance or to compare microbial community structures of two or more different samples. Therefore, the results presented hereafter in this manuscript will focus on mRNA transcripts from our ribodepleted samples.

### Taxonomic classification of mRNA transcripts

**Figure 2:**
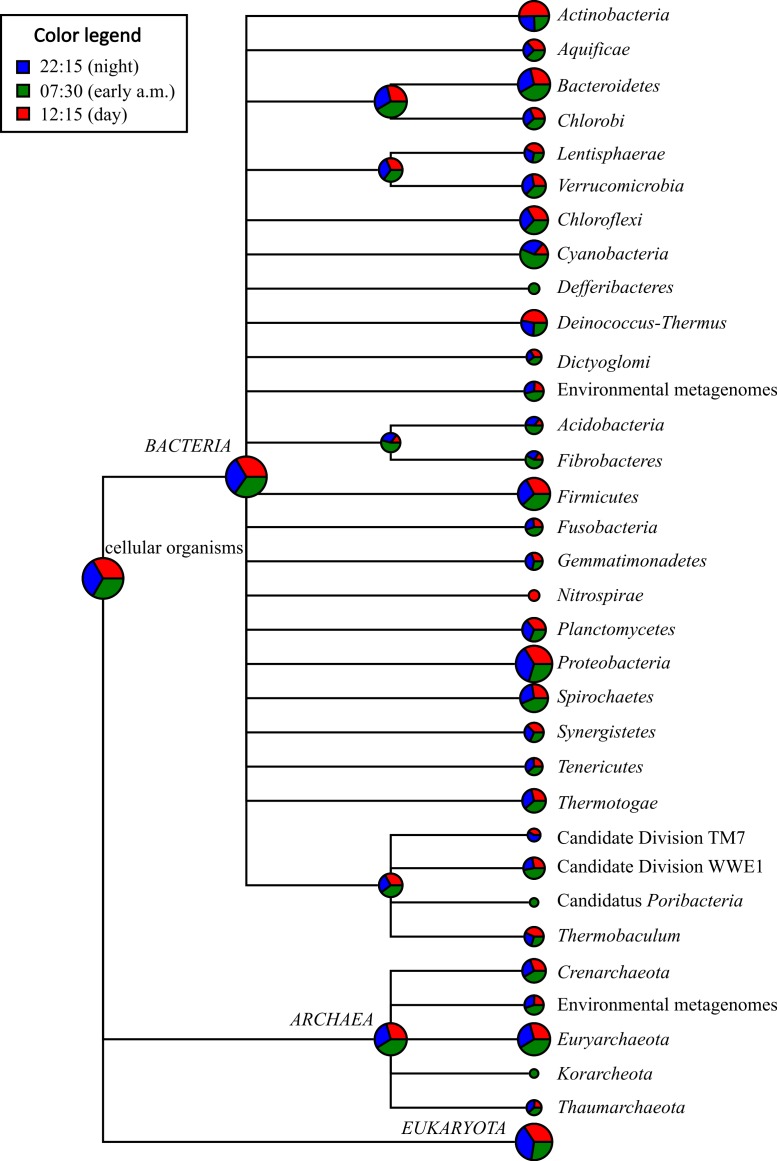
Taxonomic distribution of bacterial and archaeal mRNA transcripts in Zodletone sediment samples. Values are based on relative abundance of transcripts associated with each phylum, as determined using MEGAN 4.0, at three different time points: 22:15 (*n* = 34,835 reads), 07:30 (*n* = 53,381 reads), and 12:15 (*n* = 35,643 reads). Pie chart sizes are proportional to the overall relative abundance of each taxon. Relative abundance values representing the proportion of potential mRNA transcripts that yielded no significant BLASTX alignments for each time point are not shown. Specific relative abundance values for mRNA transcripts mapping to different taxa at the phylum-, class-, and order-levels are reported for each sample in Table S3.

On average, *Proteobacteria*-affiliated mRNA transcripts were most abundant (avg. abundance among the three potential mRNA datasets = 12.47%), followed by those affiliated with *Firmicutes* (avg. = 3.65%), *Bacteroidetes* (avg. = 3.64%), *Euryarchaeota* (avg. = 3.29%), *Actinobacteria* (avg. = 1.70%), and *Spirochaetes* (avg. = 1.25%) (Fig. 2 and Table S3). A large proportion of the mRNA transcripts were classified as unassigned *Bacteria* (avg = 9.08%) and unassigned cellular organism (avg. = 2.13%). Thus, there was a broad agreement in taxonomic assignments gleaned from mRNA datasets (Fig. 2 and Table S3) compared to the SSU rRNA control dataset described above (Fig. S2 and Table S2) and with previous SSU rRNA gene-based surveys conducted with Zodletone sediments (Elshahed et al., 2003; Youssef, Couger & Elshahed, 2010).

With respect to the phototrophic lineages, *Cyanobacteria*-affiliated mRNA transcripts were most abundant, especially in the early morning (07:30) sample, comprising 1.26% of total potential mRNA transcripts at that time (Fig. 2 and Table S3). In the afternoon (12:15), transcripts affiliated with the class *Chloroflexi* were most abundant, comprising 0.637% of total potential mRNA transcripts (Table S3). Protein-coding transcripts mapping to *Chlorobi* (green sulfur bacteria) were also detected, but were less abundant (avg abundance = 0.181%) than those mapping to *Cyanobacteria* or *Chloroflexi* (Table S3). Protein-coding transcripts that mapped to the phototrophic purple sulfur bacteria (*Chromatiales*) and the purple non-sulfur bacteria (*Rhizobiales*, *Rhodospirillales*, *Rhodobacterales*, and *Rhodocyclales*) were also detected among potential mRNA datasets at all three time points (Table S3).

With respect to lineages typically composed of aerobic chemotrophs, mRNA transcripts mapping to the predominantly aerobic chemoheterotrophic lineages *Actinobacteria* and *Halobacteria* were more highly transcribed at 12:15 (comprising 2.77 and 0.497% of total potential mRNA transcripts, respectively) compared to the other two time points (Fig. 2 and Table S3). Similarly, mRNA transcripts assigned to lineages containing aerobic sulfide/sulfur-oxidizing chemolithotrophs, such as *the Sulfurovum* group within the order *Campylobacterales* in the class *Epsilonproteobacteria* (Inagaki et al., 2004), and the order *Thiotrichales* within the *Gammaproteobacteria* (Garrity, Bell & Lilburn, 2005), were also detected at their highest relative abundance values at 12:15 (Fig. 2 and Table S3). Protein-coding transcripts mapping to aerobic type I methanotrophs, i.e., *Methylococcales* within the *Gammaproteobaceria* (Bowman, 2005), however, were approximately 5-fold more abundant at night (22:15) compared to 07:30 and 12:15 datasets (Table S3).

Protein-coding transcripts assigned to taxa typically composed of anaerobic chemotrophs, including the *Bacteroidetes*, *Spirochaetes*, *Fibrobacteres*, *Deltaproteobacteria*, and *Methanomicrobia*, were more highly represented in the early morning (07:30) dataset compared to the other two time points (Fig. 2 and Table S3). Protein-coding transcripts assigned to classes *Clostridia* and *Bacilli* within *Firmicutes* were most abundant at 12:15 (Fig. 2 and Table S3).

### Functional distribution of mRNA transcripts

**Figure 3:**
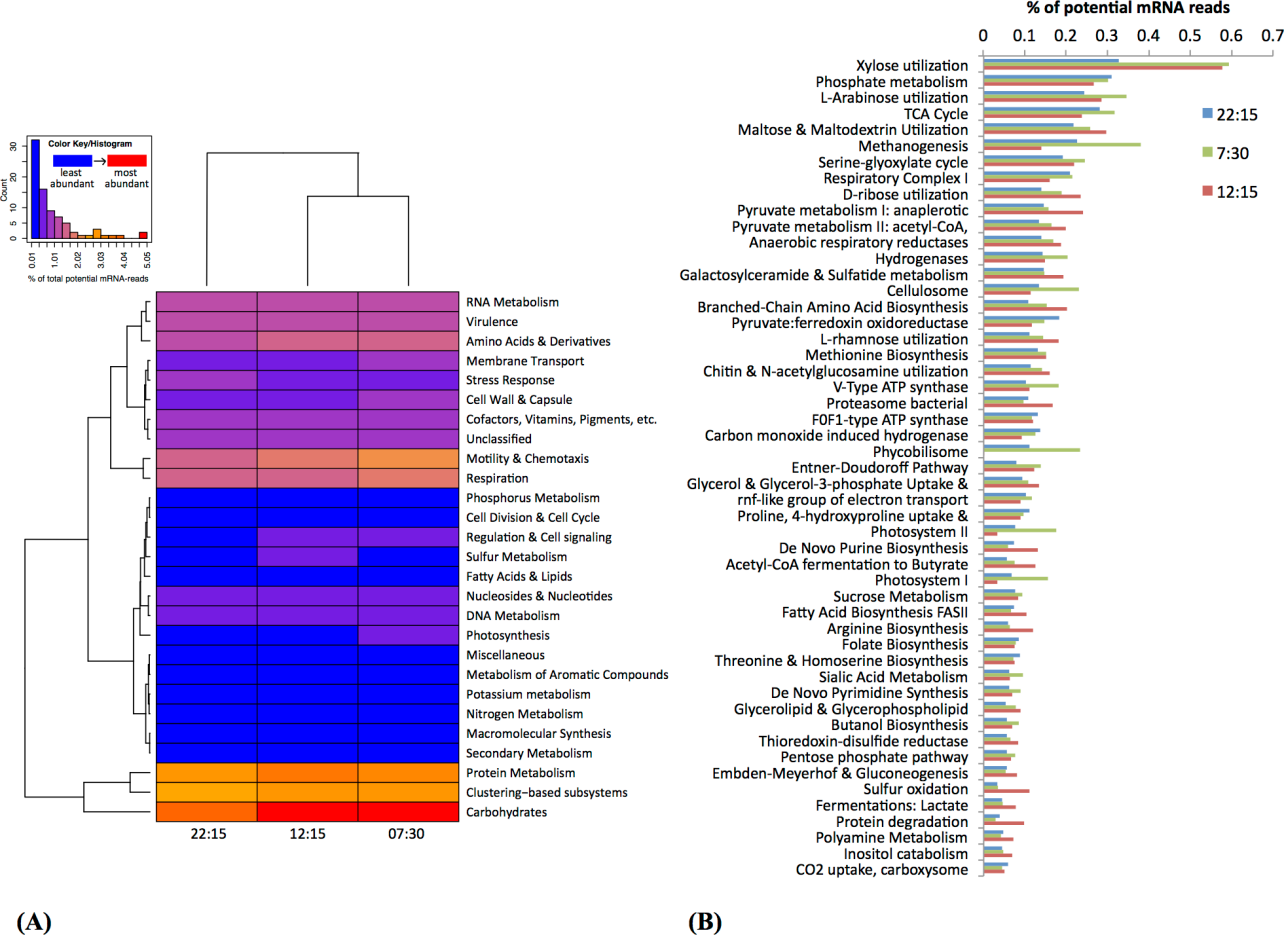
Functional classification and distribution of mRNA transcripts from Zodletone sediment samples. The heatmap (A) shows a functional-based clustering of samples based on transcript annotations into MG-Rast (v2) subsystem categories. The bar graph (B) shows the most abundant metabolic subsystems identified from Zodletone source metatranscriptomes at night (blue bars), in the early morning (green bars), and in the afternoon (pink bars).

Based on MG-RAST analysis, carbohydrate metabolism and protein metabolism were among most abundant subsystem categories detected (Fig. 3A). Specifically, xylose utilization was the most abundant among metabolic subsystems detected (Fig. 3B); utilization of xylose and other simple sugars (e.g., arabinose, maltose, and ribose) were more abundant at 07:30 and 12:15 than at 22:15 (Fig. 3B).

Subsystems related to photosynthesis, i.e., Photosystems II and I, and light-harvesting protein complexes known as phycobilisomes (De Mot et al., 1999), were most highly transcribed in the early morning (07:30) (Figs. 3A and 3B), consistent with the abundance of *Cyanobacteria*-affiliated mRNA transcripts at that time (Fig. 2 and Table S3).

**Figure 4:**
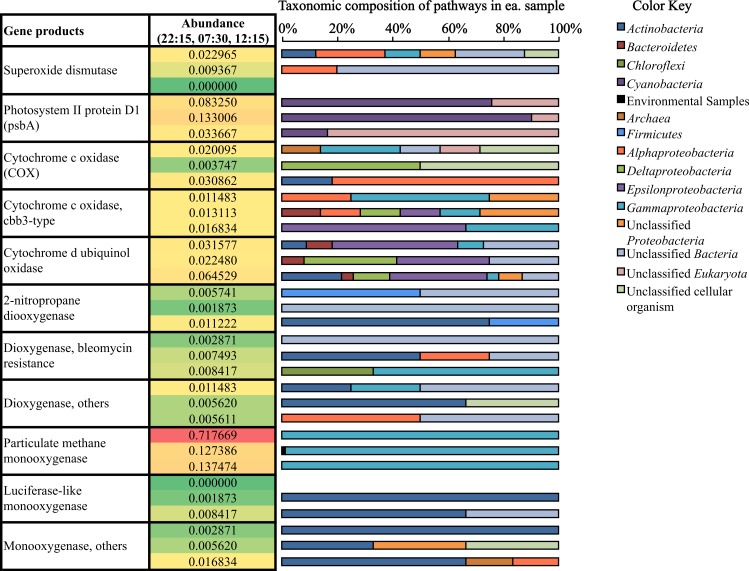
Relative abundance and taxonomic composition of mRNA transcripts involved in O_2_ production and utilization. Rel. abundance values shown are the % of potential mRNA transcripts from each sample, 22:15 (top of ea. box). 07:30 (middle), and 12:15 (bottom). The color scale ranks each gene from least abundant (green) to most abundant (red).

Transcripts coding for the copper-containing particulate methane monooxygenase, one of the two major enzymes involved in methane oxidation (Nguyen et al., 1998; Schwarz, 2001), were highly abundant at 22:15 (Fig. 4), consistent with the high relative abundance of *Methylococcales*-affiliated mRNA transcripts also observed at this time (Table S3). Methanogenesis transcripts, one the other hand, were more highly abundant in the early morning (07:30) sample (Fig. 3B), which is consistent with the higher relative abundance of *Methanomicrobia*-affiliated mRNA transcripts also detected at that time (Table S3). Genes for hydrogenases (Semrau et al., 1995) and cellulosomes, which compartmentalize cellulose degradation in anaerobes (Vignais & Billoud, 2007), were also most abundant at 07:30 (Fig. 3B).

Bacterial proteasomes, which compartmentalize protein degradation enzymes and processes inside a bacterial cell (Westley & Green, 1959), along with protein degradation subsystems were detected at a higher abundance at 12:15 compared to the other two time points (Fig. 3B). Genes for fermentation pathways (butyrate and lactate) and sulfur oxidation, also appeared to be more highly transcribed at 12:15 compared to the other two timepoints (Fig. 3B).

### Diversity and abundance of S cycling transcripts

**Figure 5:**
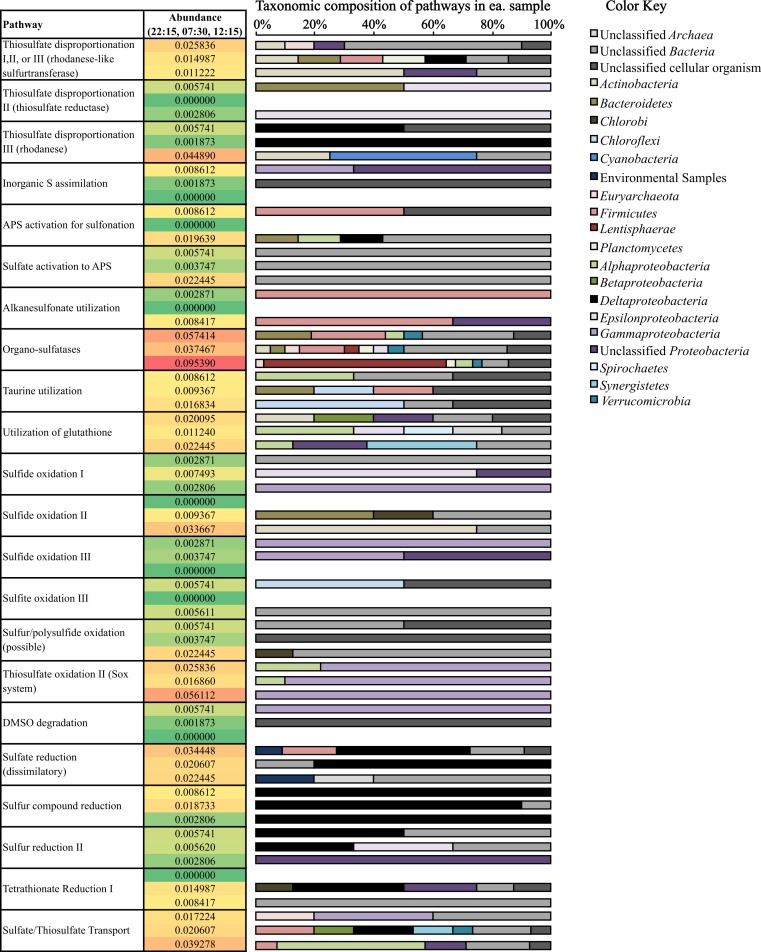
Relative abundance and taxonomic composition of sulfur cycling transcripts. Rel. abundance values shown are the % of total potential mRNA transcripts from each sample, 22:15 (top of ea. box). 07:30 (middle), and 12:15 (bottom). The color scale ranks each gene from least abundant (green) to most abundant (red). A full list of gene names categorized in each pathway can be found below Table 2.

(i) *Oxidation of sulfur compounds.* Consistent with sulfide loss occurring during the day (Fig. 1), transcripts coding for sulfide oxidation were more abundant (0.034% of potential mRNA reads, respectively) at 12:15 (Fig. 5) compared to other time periods. Surprisingly, many transcripts coding for sulfide oxidation (flavocytochrome c sulfide dehydrogenase) mapped to the phylum *Actinobacteria* (Fig. 5). A drop in thiosulfate was also observed during the day (Fig. 1); likewise, thiosulfate oxidation transcripts belonging to the Sox system were also observed at their highest abundance (0.056% of potential mRNA reads) at 12:15 (Fig. 5). A closer look at Sox genes transcribed at 12:15 showed that the all mapped to *Gammaproteobacteria* (Fig. 5); specifically, mRNA transcripts assigned as *Thiotrichales*, which includes chemolithotrophic sulfur-oxidizing bacteria (Garrity, Bell & Lilburn, 2005), comprised 65.0% of these, and those assigned as *Chromatiales*, which are the anoxygenic sulfide-oxidizing phototrophs known as the purple sulfur bacteria (Imhoff, 2005), comprised 25.0%. Gene transcripts involved in sulfur/polysulfide-oxidation processes were also more abundant at 12:15 (0.022% of potential mRNA reads) than at other time points; the vast majority of these genes expressed at all time points (91.7%) remained taxonomically unclassified (Fig. 5). *Chlorobi* (green sulfur bacteria)-affiliated transcripts only comprised a small number of sulfide and polysulfide oxidizing transcripts (Fig. 5).

(ii) *Reduction of sulfur compounds*. Consistent with patterns of sulfide production occurring overnight (Fig. 1), transcripts coding for many genes involved in pathways involving the reduction of sulfur compounds were most abundant at night (22:15) and in the early morning (07:30) (Fig. 5). *Deltaproteobacteria*- and *Firmicutes*-affiliated transcripts comprised a large proportion of dissimilatory sulfate-reduction genes, which were most highly transcribed at night (0.034% of potential mRNA reads) compared to other time points. *Deltaproteobacteria*-affilated transcripts also comprised a large majority of genes transcribed for sulfur compound reduction, which were most highly transcribed at 07:30 (0.019% of potential mRNA reads) (Fig. 5). Transcripts coding for tetrathionate reduction were also detected at their highest levels at 07:30 (0.015% of potential mRNA reads); of these, many also mapped to the *Deltaproteobacteria* (Fig. 5). Interestingly, the majority of transcripts involved in dissimilatory sulfate reduction and tetrathionate reduction detected at 12:15 were taxonomically unassigned compared to those detected either at 22:15 or 07:30 (Table 2 and Fig. 5).

**Table 2:** Distribution of the percentage of transcripts in each S cycling category, or collection of related pathways, that remained unclassified at the phylum-level.

Sample/time point	No. S cycling reads	% of S cycling reads unclassified	% unclassified (phylum-level) in each S-cycling pathway					
			Organic S metabolism^a^	Inorganic S assimilation^b^	Reduction^c^	Oxidation^d^	Disproportionation^e^	Transport^f^
22:15	94	41.5	45.2	62.5	27.8	27.8	61.5	33.3
07:30	114	31.6	43.8	100.0	26.5	20.8	22.2	18.2
12:15	160	34.4	25.5	75.0	64.3	27.3	23.8	28.6
Average		35.8	38.1	79.2	39.5	25.3	35.9	26.7
StDev		5.1	11.0	19.1	21.5	3.9	22.3	7.7

**Notes.**

a Organic S metabolisms pathways (and genes) include: alkanesulfonate utilization (alkanesulfonates binding protein); Organosulfatases (sulfatase); Taurine utilization (Gamma-glutamyl-transpeptidase, Taurine transport protein TauB, Taurine-pyruvate aminotransferase, and Taurine dioxygenase TauD); glutathione utilization (putative glutathione transporter).

b Inorganic S assimilation pathways (and genes) include: inorganic S assimilation (3’(2’)5’-bisphosphate nucleotidase); sulfate activation to APS (assimilatory-type sulfate adenylyltransferase); APS activation for sulfonation (APS kinase); assimilatory sulfate reduction (assimilatory-type sulfite reductase).

c Reduction pathways (and genes) include: DMSO degradation (DMSO reductase); dissimilatory sulfate reduction (dissimilatory-type sulfate adenylyltransferase, APS reductase, dissimilatory sulfite reductase); sulfur reduction I (H_2_:sulfur or NADH:sulfur oxidoreductase); Sulfur reduction II (polysulfide reductase), and tetrathionate reduction (tetrathionate reductase).

d Oxidation pathways (and genes) include: sulfide oxidation I (sulfide:quinone oxidoreductase); sulfide oxidation II (flavocytochrome c sulfide dehydrogenase); sulfide oxidation III (reverse-type sulfite reductase); sulfite oxidation I (sulfite:cytochrome c oxidoreductase); sulfite oxidation II (reverse-type APS reductase), sulfite oxidation III (sulfite oxidase); possible sulfur/polysulfide oxidation (NADH oxidase/NADH:polysulfide oxidoreductase); thiosulfate/sulfur oxidation/Sox operon (SoxA, SoxB, SoxC, SoxD, SoxH, SoxX, SoxY).

e Disproportionation pathways (and genes) include: thiosulfate disproportionation I,II, or III (rhodanese-like sulfurtransferase), thiosulfate disproportionation I or II (thiosulfate reductase); thiosulfate disproportionation II/cyanate pathway (rhodanese, cyanate hydratase).

f Transport genes include: ABC-type nitrate/sulfonate/bicarbonate transporter; sulfate & thiosulfate binding protein CysP; sulfate & thiosulfate import protein CysA; sulfate & thiosulfate permease protein CysT; sulfate permease; Trk-type sulfate permease; sulfate transporter CysZ.

(iii) *Thiosulfate disproportionation*. Transcripts for rhodanese, a thiosulfate sulfurtransferase that reacts thiosulfate with cyanide producing thiocyanate (Westley & Green, 1959; Pagani et al., 1991; Bordo et al., 2000), were most abundant at 12:15 (0.045% of potential mRNA reads) (Fig. 5). Rhodanese transcripts detected at this time mapped to *Actinobacteria* and *Cyanobacteria*, whereas those detected at 22:15 and 07:30 were predominantly affiliated with *Deltaproteobacteria* (Fig. 5). Rhodanese-like sulfurtransferases, which may be involved in thiosulfate disproportionation among other possible types of sulfur metabolism (Cipollone, Ascenzi & Visca, 2007) were most abundant at 22:15 (0.026% of potential mRNA reads); a large proportion of these genes remained taxonomically unclassified (Fig. 5). Thiosulfate reductase, also involved in thiosulfate disproportionation (Haschke & Campbell, 1971; Aketagawa, Kobayashi & Ishimoto, 1985), was not as abundant compared to the other two transcripts mentioned above (Fig. 5).

(iv) *Organic sulfate metabolism*. Organo-sulfatase transcripts (Fig. 5), which code for enzymes that release sulfate through the breakdown of sulfate ester compounds (Kertesz, 1999), were the most abundant of any S-cycling gene at 12:15 (0.095% of potential mRNA reads) and were also fairly abundant at the other two time points as well (0.057% and 0.037% of potential mRNA reads at 22:15 and 07:30, respectively) (Fig. 5). A broad diversity of bacterial phyla, including *Lentisphaerae* and *Firmicutes*, transcribed these genes, but many also remained unclassified (Fig. 5). In fact, a high proportion of genes transcribed for organic S metabolism (25.5–43.8%) and assimilation of inorganic sulfur into organic matter (62.5–100%) remained taxonomically unclassified at the phylum- or domain-level (Table 2 and Fig. 5).

### Expression of genes coding for O_2_ production and utilization processes in source sediment

Mining our potential mRNA data subsets for reads and assembled contigs involved in oxygen cycling revealed the diversity of microbes participating in both oxygen production and utilization in Zodletone source sediment (Fig. 4). *Cyanobacteria*, which were previously considered anoxygenic in the source (Bühring et al., 2011), showed active transcription of the *psbA* gene (Fig. 4), which codes for the oxygen-producing P680 reaction center D1 protein in Photosystem II (Zouni et al., 2001; Kamiya & Shen, 2003; Ferreira et al., 2004). In addition to cyanobacterial genes, eukaryotic *psbA* genes (classified as “unclassified Eukaryota” in Fig. 4) had top BlastX hits to *psbA* genes found among different diatom genomes, including *Thalassiosira pseudonana*, *Odontella sinensis*, and *Phaeodactylum tricornutum*. Overall, the relative abundance of *psbA* transcripts was greatest at 07:30 (0.133% of potential mRNA reads) (Fig. 4). Alternatively, transcripts coding for superoxide dismutase, which also catalyze the production of O_2_, were most abundant at 22:15 (Fig. 4).

Oxygen-utilization genes were diverse in type and in taxonomic distribution (Fig. 4). Transcripts for high-affinity terminal oxidases (cbb3-type cytochrome c oxidase and cytochrome d ubiquinol oxidase) as well as typical cytochrome c oxidases (COX) were transcribed at their highest levels at 12:15 (Fig. 4). As well, several monooxygenase and dioxygenase gene transcripts were detected (Fig. 4). Gene coding for the particulate methane monooxygenase, which uses O_2_ in the oxidation of methane to methanol during methane oxidation (Nguyen et al., 1998; Schwarz, 2001), were most highly transcribed at 22:15 (0.718% of potential mRNA reads) (Fig. 4), and all mapped to the order *Methylococcales*, which includes Type I methanotrophs within the *Gammaproteobacteria* (Bowman, 2005). Overall, transcripts for oxygen utilization were least abundant at 07:30 (Fig. 4).

## Discussion

In this study, we investigated the nature and dynamics of predominant microbial taxa and metabolic processes occurring at Zodletone Spring through the analysis of mRNA transcripts expressed in sediments following sunrise (early morning), at noon, and at night. The study provides insight into a complex sulfur cycle that includes the possible involvement of *Actinobacteria* in sulfur transformations and identifies several interesting features not previously documented in the anoxic sediments of this high-sulfide sunlight-exposed aquatic spring, including a full methane cycle, with activity of Type I methanotrophs at night and the likelihood of O_2_ production and utilization through oxygenic photosynthesis and aerobic metabolism, respectively.

Mining of metatranscriptomic datasets for gene transcripts coding for sulfur cycling processes indeed highlighted a complex sulfur cycle in Zodletone sediments. Briefly, sulfide and thiosulfate loss observed during the day (Fig. 1) could be attributed to both phototrophic and aerobic chemotrophic oxidation processes carried out by *Chromatiales*, *Thiotrichales*, and *Actinobacteria* (Fig. 5). Similarly, sulfidogenesis that occurs overnight (Fig. 1) could be a function of multiple processes carried out in large part by *Deltaproteobacteria*, including sulfate reduction, sulfur reduction, thiosulfate disproportionation, and tetrathionate reduction (Fig. 5).

Evidence for aerobic sulfide and thiosulfate metabolism by *Actinobacteria* is also presented in this study (Fig. 5). Specifically, many of the transcripts encoding sulfide oxidation (flavocytochrome c sulfide dehydrogenase) and thiosulfate disproportionation (rhodanese) genes detected during the day (12:15) were mapped to the phylum *Actinobacteria* (Fig. 5). As taxonomic analyses of mRNA transcripts also show that *Actinobacteria*-affiliated mRNA transcripts were at their highest levels during the day (Fig. 2 and Table S3), and because many terminal cytochrome oxidase genes transcribed during the day also mapped to the phylum *Actinobacteria* (Fig. 4), it is also possible that these processes are supported by an aerobic mode of metabolism. Some species of *Actinobacteria* have been shown to be involved in aerobic oxidation of dimethylsulfide and dimethyldisulfide (Reichert et al., 1998) as well as in the oxidation of sulfur and pyrite under acidic conditions (Norris et al., 2011). Additionally, other species of *Actinobacteria*, *Microbacterium phyllosphaerae* and *Leifsonia shinshuensis*, were recently isolated from rhizosphere soils that are capable of the oxidation of thiosulfate, tetrathionate, trithionate and sulfur (Anandham et al., 2008). Both of these species contained a variety of genes involved in sulfur oxidation including rhodanese, thiosulfate oxidase and sulfite oxidase (Anandham et al., 2008).

These metatranscriptomic data also revealed that the production of sulfate in source sediments may not occur solely through inorganic sulfur metabolism, i.e., phototrophic sulfide/sulfur oxidation. Organo-sulfatase transcripts were much more abundant at all three time points compared to transcripts that encode for the complete oxidation of sulfur compounds to sulfate (Fig. 5). As well, organo-sulfatase transcripts were approx. twice as abundant as those that encode thiosulfate transformation (Fig. 5), another possible sulfate source. The role of sulfatases in *Bacteria* is typically in the cleavage of sulfate esters of organic compounds, either providing a source of sulfate or a source of organic carbon (Hanson, Best & Wong, 2004). As free sulfate is not likely a limiting nutrient in this system, the abundance of sulfatase transcripts indicates the relative importance of sulfonated organic compounds as nutrients in the spring. Together, the detection of sulfatase and sulfonation transcripts (Fig. 5) suggests that sulfate cycling includes organic sulfonated compounds within the source sediments.

Based on patterns observed from general subsystems and specific metabolic transcripts (Figs. 3A, 3B and 4) along with the shifts in taxonomic lineages observed in potential mRNA datasets (Fig. 2), we reason that the transcriptional activities of several members of the microbial community are linked to specific metabolic processes occurring during the day or night. For example, the abundance of methanogenesis functional gene transcripts (Fig. 3B) and transcripts mapping to methanogenic taxa in the early morning (Fig. 2) and the surprisingly high abundance at night of *Methylococcales*-affiliated transcripts and those coding for the particulate methane monooxygenase (Fig. 4), suggests that a complete methane cycle is occurring in Zodletone source sediment. *Methylococcales*, which has previously not been considered a dominant member in the Zodletone microbial community studies analyzing SSU rRNA genes during the daytime only (Elshahed et al., 2003; Youssef, Couger & Elshahed, 2010), may thus play a key role in the cycling of methane or other C1 compounds in Zodletone source sediments.

Despite the history of a lack of O_2_ in Zodletone source sediments (Senko et al., 2004; Bühring et al., 2011), an abundance of aerobic taxa and aerobic respiration genes were detected among potential mRNA datasets (Figs. 2 and 4); these observations could be explained if oxygenic phototrophs supported the growth and metabolism of aerobic or microaerophilic taxa, a relationship that has not been previously documented under the sunlit, high-sulfide conditions that predominate at Zodletone. In this study, gene transcripts encoding oxygenic photosynthetic processes were indeed expressed by *Cyanobacteria* and diatoms (Fig. 4), suggesting the possibility of O_2_ evolution under these conditions. While *Cyanobacteria* are typically considered oxygenic phototrophs, anoxygenic phototrophy has been documented among members of this phylum as an alternative to, or even replacement for, oxygenic phototrophy (Cohen, Padan & Shilo, 1975; Belkin & Padan, 1978; Jorgensen, Cohen & Revsbech, 1986; Voorhies et al., 2012). It has also been suggested that photosynthesis in *Cyanobacteria* will be entirely anoxygenic at sulfide concentrations >1 mM (Cohen et al., 1986). Because sulfide concentrations in Zodletone source water far exceed this threshold, ranging between 6.5–9 mM (Fig. 1), and a previous suggestion (based on the lack of detectable O_2_ at the site during the daytime) that anoxygenic photosynthesis was the primary metabolic mode for *Cyanobacteria* in Zodletone source sediment (Bühring et al., 2011), it is surprising, here, that we report the possible production of O_2_ under high-sulfide concentrations based on the transcription of cyanobacterial *psbA* genes, which code for the oxygen-producing subunit of Photosystem II Protein D1 (Zouni et al., 2001; Kamiya & Shen, 2003; Ferreira et al., 2004). Furthermore, it appears that this O_2_, likely produced through oxygenic photosysnthesis (Fig. 4), could be an important electron acceptor in Zodletone source sediment (Fig. 4), in spite of the highly reduced conditions present, analogous to the recently described microorganisms that can produce O_2_through nitrite reduction and then use the O_2_ for methane oxidation under anoxic conditions (Ettwig et al., 2010). Because oxygen levels in the source sediment are below detectable levels, these data could suggest a yet-unidentified mechanism for oxygen transfer between oxygenic phototrophs and aerobic chemotrophs or a rapid consumption of produced O_2_, preventing its accumulation to a detectable level. While the former has not been documented in the literature, the latter has been observed in marine sediments, but with maximum rates of O_2_ production exceeding those of O_2_ consumption in photic zones, resulting in low steady-state O_2_ concentrations (Revsbech, Madsen & Jorgensen, 1986) rather than O_2_ being below detectable levels, as seen in Zodletone sediments (Senko et al., 2004; Bühring et al., 2011). However, the high sulfide concentrations at Zodletone could both greatly limit the levels of oxygenic photosynthesis to far smaller rates than observed in marine sediments or react abiotically with O_2_ produced to form pentasulfide or thiosulfate, as observed in an alkaliphilic bacterial sulfoxidizing consortium (González-Sánchez & Revah, 2007).

That the O_2_ available for sulfide oxidation may be derived from oxygenic photosynthesis rather than from the atmosphere or sub-oxic waters overlaying source sediments is supported by previous studies at Zodletone that find that rates of sulfide loss are most rapid in light-exposed sediments, even in aerobic incubations exposed to O_2_ (Elshahed et al., 2003; Senko et al., 2004). Previous interpretations of these studies were that sulfide oxidation was driven by anoxygenic photosynthesis; the data presented here, however, also lends support to the possibility that sulfide oxidation in sunlight-exposed high sulfide environments could also be an aerobic chemolithotrophic process supported by O_2_ produced from oxygenic phototrophs.

## Conclusion

The use of metatranscriptomics for describing complex microbial ecosystems such as Zodletone spring has its limitations, including the probable occurrence of errors in functional and taxonomic assignment of functional genes. Also, the assumption involved in correlating transcript abundance with protein abundance/metabolic activity must always be taken with caution, as transcriptional and translational activity can differ in some cases (Gygi et al., 1999; Suzuki, Simon & Slabas, 2006). Despite these limitations, however, this study implicates several interesting microbial processes and interactions occurring in this sunlit, shallow, sulfur spring system, including a full sulfur and methane cycle that involve both aerobic and anaerobic bacteria in an anoxic sediment system, with O_2_ that is likely produced by *Cyanobacteria* and diatoms by oxygenic photosynthesis. Lastly, the production of sulfate from mechanisms other than anoxygenic photosynthesis, such as aerobic sulfide and thiosulfate metabolism as well as the cleavage of sulfate esters by sulfatases, sheds insight into the microbial metabolism and lifestyles on early earth, prior to the oxygenation of the atmosphere. This work may present alternative explanations for the increase in sulfate concentrations that stimulated sulfate reduction as oxygen began to first accumulate in the atmosphere during the late Archaean and Proterozoic eons (Canfield, 1998; Reinhard et al., 2009).

## Supplemental Information

10.7717/peerj.1259/supp-1 Figure S1 Zodletone spring and Saddle Mountain Creek in southwestern Oklahoma Sediment samples were taken for RNA extraction and processing from the south-facing corner of Zodletone spring (A). Downstream of where the spring’s stream enters Saddle Mountain Creek (B) shows a vibrant phototrophic plume. Image obtained from Google Earth © 2015.

10.7717/peerj.1259/supp-2 Figure S2 SSU rRNA-based microbial community composition from datasets obtained from non-ribodepleted RNA (left) vs. ribodepleted RNA (right)

10.7717/peerj.1259/supp-3 Table S1 Analysis of SSU rRNA datasets obtained from Zodetone source sediment metatranscriptomes and comparison of taxonomic lineages detected from non-ribodepleted vs. ribodepleted RNA datasets Metatranscriptome datasets for both non-ribodepleted RNA and ribodepleted samples were derived from the same original RNA sample (12:15)–pre- and and post-rRNA removal, respectively.

10.7717/peerj.1259/supp-4 Table S2 Taxonomic lineages (phylum-, class-, and order-levels) detected from Zodletone SSU rRNA data subsets Values shown are from metatranscriptomic libraries created from night (22:15), early morning (07:15) and afternoon (12:15) samples ribodepleted samples, as well as from the non-ribodepleted 12:15 sample.

10.7717/peerj.1259/supp-5 Table S3 Taxonomic lineages (phylum-, class-, and order-levels) detected from Zodletone potential mRNA data subsets Values shown represent relative abundance values of taxa from metatranscriptomic libraries created from night (22:15), early morning (07:15) and afternoon (12:15) ribodepleted samples.

## Additional Information and Declarations

### DNA Deposition

The following information was supplied regarding the deposition of DNA sequences: Genbank (SRX025760, SRX025825, SRX025796, and SRX331735).

### Data Availability

The following information was supplied regarding data availability: MG-RAST (4450335.3, 4450336.3, 4450338.3, 4451038.3, 4451039.3, 4451037.3, 4453253.3, 4453260.3, 4453251.3, and 4451718.3).
