# Supplementary material for: Metatranscriptomic analysis of a high-sulfide aquatic spring reveals insights into sulfur cycling and unexpected aerobic metabolism
Source: PeerJ. 2015 Sep 22;3:e1259. doi: 10.7717/peerj.1259 (PMC4582958; doi:10.7717/peerj.1259)
Supplement: Figure S1 — Sediment samples were taken for RNA extraction and processing from the south-facing corner of Zodletone spring (A). Downstream of where the spring’s stream enters Saddle Mountain Creek (B) shows a vibrant phototrophic plume. Image obtained from Google Earth © 2015. [file peerj-03-1259-s001.pdf]

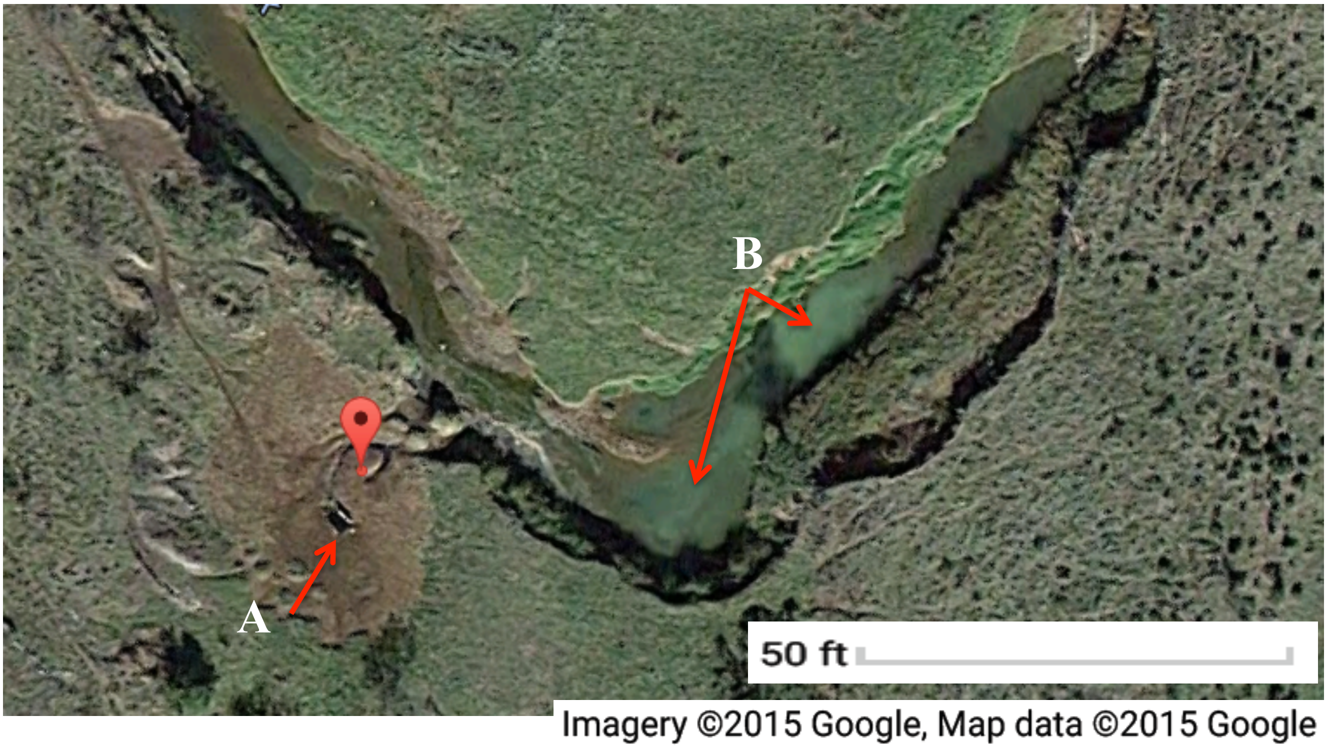

Supplemental Figure 1. South-facing corner of Zodletone Spring (A), where four sediment samples were taken for RNA extraction and processing. Downstream of where the spring's stream enters Saddle Mountain Creek (B) shows a vibrant phototrophic plume. Image obtained from Google Earth ©2015.
