# Supplementary material for: Metatranscriptomic analysis of a high-sulfide aquatic spring reveals insights into sulfur cycling and unexpected aerobic metabolism
Source: PeerJ. 2015 Sep 22;3:e1259. doi: 10.7717/peerj.1259 (PMC4582958; doi:10.7717/peerj.1259)
Supplement: Figure S2 [file peerj-03-1259-s002.pdf]

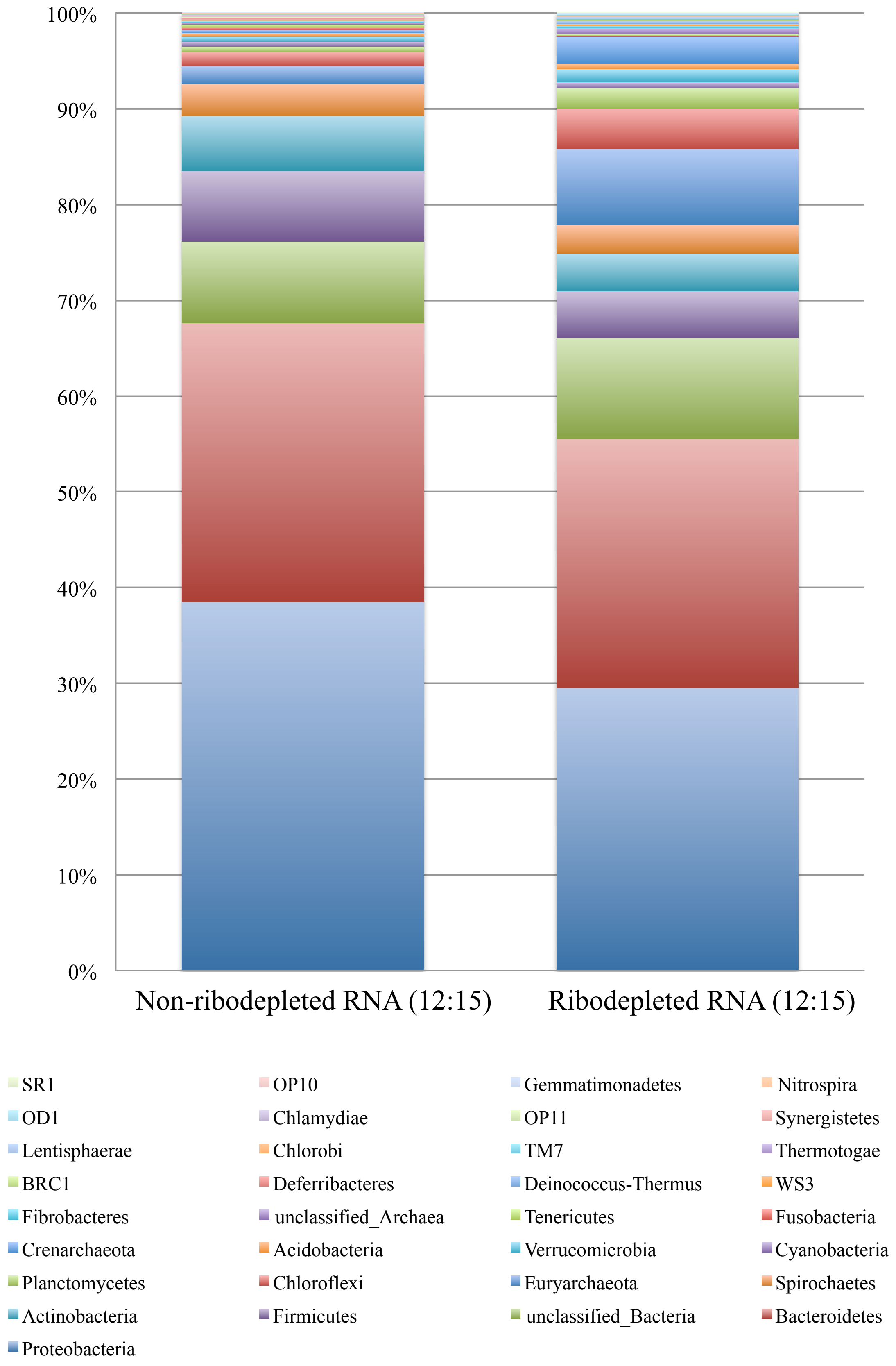

Supplemental Figure 2. SSU rRNA-based microbial community composition from datasets obtained from non-ribodepleted RNA (left) vs. ribodepleted RNA (right).
