# Supplementary material for: Metatranscriptomic analysis of a high-sulfide aquatic spring reveals insights into sulfur cycling and unexpected aerobic metabolism
Source: PeerJ. 2015 Sep 22;3:e1259. doi: 10.7717/peerj.1259 (PMC4582958; doi:10.7717/peerj.1259)
Supplement: Table S1 — Metatranscriptome datasets for both non-ribodepleted RNA and ribodepleted samples were derived from the same original RNA sample (12:15)–pre- and and post-rRNA removal, respectively. [file peerj-03-1259-s003.docx]

**Supplemental Table 1.**  Analysis of SSU rRNA datasets obtained from Zodetone source sediment metatranscriptomes prepared from ribo-depleted RNA and comparison of taxonomic lineages detected from rRNA-depleted vs. total RNA datasets. Metatranscriptome datasets for both total RNA and rRNA-depleted samples were derived from the same original RNA sample (12:15) – pre- and and post-rRNA removal, respectively.

| Sample/time of day | No. of SSU rRNA^1^ reads | No. unclassified^2^ (%) | No. belonging to candidate divisions (%) | No. Taxonomic Lineages Detected | | |
| --- | --- | --- | --- | --- | --- | --- |
|  |  |  |  | Phyla | Classes | Orders |
| 22:15 | 26,410 | 3000 (11.4%) | 312 (1.2%) | 31 | 77 | 144 |
| 07:30 | 23,273 | 3104 (13.4%) | 224 (0.96%) | 33 | 76 | 145 |
| 12:15 | 26,791 | 2982 (11.1%) | 142 (0.53%) | 31 | 75 | 140 |
| 12:15 (non-ribodepleted RNA control) | 19,729 | 1717 (8.7%) | 73 (0.37%) | 31 | 71 | 133 |

^1^Microbial SSU rRNA reads only (*Bacteria* and *Archaea*) as determined by classification using the RDP classifier program.

^2^Unassigned taxonomically at the phylum level.
