# Supplementary material for: Metatranscriptomic analysis of a high-sulfide aquatic spring reveals insights into sulfur cycling and unexpected aerobic metabolism
Source: PeerJ. 2015 Sep 22;3:e1259. doi: 10.7717/peerj.1259 (PMC4582958; doi:10.7717/peerj.1259)
Supplement: Table S2 — Values shown are from metatranscriptomic libraries created from night (22:15), early morning (07:15) and afternoon (12:15) samples ribodepleted samples, as well as from the non-ribodepleted 12:15 sample. [file peerj-03-1259-s004.docx]

**Supplemental Table 2**. Taxonomic lineages (phylum-, class-, and order-levels) detected from Zodletone SSU rRNA datasets from within metatranscriptomic libraries created from night (22:15), early morning (07:15) and afternoon (12:15) samples.

| Lineage (RDP taxonomy):  **Phylum**  Class/Subclass  Order | Relative abundance in each sample (%) | | | |
| --- | --- | --- | --- | --- |
|  | 22:15 | 07:30 | 12:15 | 12:15 (total RNA control) |
| ***Proteobacteria*** | **47.497** | **26.885** | **29.480** | **38.600** |
| *Gammaproteobacteria* | 29.909 | 11.094 | 10.291 | 14.938 |
| *Methylococcales* | 20.345 | 3.902 | 2.352 | 3.553 |
| unclassified*_Gammaproteobacteria* | 2.336 | 1.706 | 1.437 | 2.721 |
| *Chromatiales* | 1.844 | 1.465 | 1.736 | 2.238 |
| *Thiotrichales* | 1.492 | 0.563 | 0.668 | 0.939 |
| *Oceanospirillales* | 1.420 | 1.590 | 1.635 | 1.888 |
| *Alteromonadales* | 1.401 | 1.074 | 1.418 | 2.172 |
| *Gammaproteobacteria_*incertae_sedis | 0.712 | 0.339 | 0.328 | 0.442 |
| *Xanthomonadales* | 0.186 | 0.254 | 0.295 | 0.477 |
| *Pseudomonadales* | 0.129 | 0.155 | 0.336 | 0.355 |
| *Enterobacteriales* | 0.030 | 0.026 | 0.030 | 0.036 |
| *Acidithiobacillales* | 0.015 | 0.013 | 0.019 | 0.061 |
| *Legionellales* | 0.000 | 0.004 | 0.015 | 0.030 |
| *Salinisphaerales* | 0.000 | 0.000 | 0.004 | 0.020 |
| *Vibrionales* | 0.000 | 0.004 | 0.019 | 0.000 |
| *Alphaproteobacteria* | 7.300 | 5.087 | 3.133 | 9.644 |
| *Rhodobacterales* | 2.972 | 2.398 | 2.934 | 3.776 |
| *Rhodospirillales* | 2.086 | 0.885 | 1.191 | 2.624 |
| *Rhizobiales* | 0.878 | 0.748 | 1.090 | 1.436 |
| *unclassified_Alphaproteobacteria* | 0.647 | 0.249 | 0.373 | 0.640 |
| *Sphingomonadales* | 0.420 | 0.580 | 0.392 | 0.929 |
| *Caulobacterales* | 0.106 | 0.142 | 0.078 | 0.102 |
| *Parvularculales* | 0.102 | 0.026 | 0.015 | 0.071 |
| *Alphaproteobacteria_*incertae_sedis | 0.064 | 0.030 | 0.056 | 0.061 |
| *Kordiimonadales* | 0.011 | 0.004 | 0.000 | 0.000 |
| *Kiloniellales* | 0.008 | 0.026 | 0.000 | 0.005 |
| *Sneathiellales* | 0.004 | 0.000 | 0.000 | 0.000 |
| *Rickettsiales* | 0.000 | 0.000 | 0.004 | 0.000 |
| *Deltaproteobacteria* | 6.619 | 7.739 | 7.936 | 9.111 |
| *Desulfuromonadales* | 2.416 | 3.115 | 3.143 | 3.624 |
| *Myxococcales* | 1.605 | 1.233 | 1.277 | 0.837 |
| *Desulfobacterales* | 1.348 | 1.315 | 1.504 | 2.314 |
| unclassified*_Deltaproteobacteria* | 0.931 | 1.345 | 1.433 | 1.507 |
| *Syntrophobacterales* | 0.220 | 0.593 | 0.437 | 0.711 |
| *Desulfovibrionales* | 0.057 | 0.064 | 0.097 | 0.030 |
| *Bdellovibrionales* | 0.038 | 0.056 | 0.034 | 0.000 |
| *Desulfarculales* | 0.004 | 0.000 | 0.011 | 0.081 |
| *Syntrophorhabdaceae* | 0.000 | 0.017 | 0.000 | 0.000 |
| *Epsilonproteobacteria* | 1.780 | 0.924 | 2.807 | 2.452 |
| *Campylobacterales* | 1.746 | 0.920 | 2.781 | 2.406 |
| unclassified*_Epsilonproteobacteria* | 0.023 | 0.004 | 0.026 | 0.041 |
| *Nautiliales* | 0.011 | 0.000 | 0.000 | 0.005 |
| Unclassified *Proteobacteria* | 1.473 | 1.353 | 1.668 | 1.675 |
| *Betaproteobacteria* | 0.417 | 0.687 | 0.646 | 0.782 |
| *Burkholderiales* | 0.310 | 0.541 | 0.556 | 0.553 |
| *Rhodocyclales* | 0.042 | 0.060 | 0.049 | 0.107 |
| *Hydrogenophilales* | 0.019 | 0.052 | 0.011 | 0.046 |
| *unclassified_Betaproteobacteria* | 0.019 | 0.004 | 0.015 | 0.061 |
| *Methylophilales* | 0.015 | 0.013 | 0.004 | 0.010 |
| *Neisseriales* | 0.008 | 0.004 | 0.004 | 0.000 |
| *Nitrosomonadales* | 0.004 | 0.013 | 0.007 | 0.005 |
| ***Bacteroidetes*** | **19.012** | **28.084** | **26.057** | **29.119** |
| *Bacteroidia* | 5.967 | 7.498 | 7.674 | 6.751 |
| *Bacteroidales* | 5.967 | 7.498 | 7.674 | 6.751 |
| unclassified*_"Bacteroidetes"* | 5.309 | 7.507 | 9.305 | 8.669 |
| *Flavobacteria* | 4.089 | 7.554 | 4.278 | 6.806 |
| *Flavobacteriales* | 4.089 | 7.554 | 4.278 | 6.806 |
| *Sphingobacteria* | 2.045 | 2.973 | 3.106 | 4.385 |
| *Sphingobacteriales* | 2.045 | 2.973 | 3.106 | 4.385 |
| *Bacteroidetes_*incertae_sedis | 1.602 | 2.552 | 1.695 | 2.507 |
| **unclassified*_Bacteria*** | **10.973** | **12.641** | **10.492** | **8.527** |
| ***Euryarchaeota*** | **4.207** | **8.770** | **7.928** | **1.870** |
| *Methanomicrobia* | 1.958 | 4.348 | 4.849 | 0.980 |
| *Methanosarcinales* | 1.166 | 2.999 | 3.718 | 0.802 |
| *Methanomicrobiales* | 0.625 | 0.932 | 0.773 | 0.102 |
| unclassified*_"Methanomicrobia"* | 0.159 | 0.417 | 0.358 | 0.076 |
| *Methanocellales* | 0.008 | 0.000 | 0.000 | 0.000 |
| unclassified*_"Euryarchaeota"* | 1.867 | 3.644 | 2.538 | 0.802 |
| *Halobacteria* | 0.326 | 0.657 | 0.347 | 0.071 |
| *Halobacteriales* | 0.326 | 0.657 | 0.347 | 0.071 |
| *Thermoplasmata* | 0.027 | 0.043 | 0.090 | 0.015 |
| *Thermoplasmatales* | 0.027 | 0.043 | 0.090 | 0.015 |
| *Methanobacteria* | 0.019 | 0.021 | 0.030 | 0.000 |
| *Methanobacteriales* | 0.019 | 0.021 | 0.030 | 0.000 |
| *Methanococci* | 0.011 | 0.043 | 0.063 | 0.000 |
| *Methanococcales* | 0.011 | 0.043 | 0.063 | 0.000 |
| *Archaeoglobi* | 0.000 | 0.009 | 0.011 | 0.000 |
| *Archaeoglobales* | 0.000 | 0.009 | 0.011 | 0.000 |
| *Methanopyri* | 0.000 | 0.004 | 0.000 | 0.005 |
| *Methanopyrales* | 0.000 | 0.004 | 0.000 | 0.005 |
| ***Chloroflexi*** | **3.090** | **3.394** | **4.195** | **1.457** |
| *Anaerolineae* | 2.204 | 2.488 | 3.008 | 0.934 |
| *Anaerolineales* | 2.204 | 2.488 | 3.008 | 0.934 |
| unclassified*_"Chloroflexi"* | 0.405 | 0.374 | 0.556 | 0.249 |
| *Thermomicrobia* | 0.261 | 0.202 | 0.216 | 0.112 |
| *Sphaerobacterales* | 0.246 | 0.189 | 0.190 | 0.112 |
| *Thermomicrobiales* | 0.008 | 0.004 | 0.004 | 0.000 |
| unclassified*_Thermomicrobia* | 0.008 | 0.009 | 0.022 | 0.000 |
| *Caldilineae* | 0.155 | 0.172 | 0.306 | 0.096 |
| *Caldilineales* | 0.155 | 0.172 | 0.306 | 0.096 |
| *Dehalococcoidetes* | 0.057 | 0.146 | 0.101 | 0.066 |
| *Dehalogenimonas* | 0.057 | 0.146 | 0.101 | 0.066 |
| *Chloroflexi* | 0.008 | 0.013 | 0.007 | 0.000 |
| *Chloroflexales* | 0.008 | 0.009 | 0.007 | 0.000 |
| unclassified_"*Chloroflexi*" | 0.000 | 0.004 | 0.000 | 0.000 |
| ***Firmicutes*** | **2.919** | **3.609** | **4.946** | **7.405** |
| *Clostridia* | 1.946 | 2.209 | 3.318 | 5.329 |
| *Clostridiales* | 1.530 | 1.822 | 2.930 | 4.954 |
| unclassified*_"Clostridia"* | 0.386 | 0.348 | 0.362 | 0.350 |
| *Halanaerobiales* | 0.015 | 0.004 | 0.007 | 0.015 |
| *Thermoanaerobacterales* | 0.015 | 0.034 | 0.019 | 0.010 |
| unclassified*_"Firmicutes"* | 0.477 | 0.752 | 0.590 | 0.528 |
| *Bacilli* | 0.470 | 0.580 | 0.970 | 1.431 |
| *Bacillales* | 0.417 | 0.503 | 0.888 | 1.325 |
| *Lactobacillales* | 0.045 | 0.073 | 0.067 | 0.096 |
| unclassified*_"Bacilli"* | 0.008 | 0.004 | 0.015 | 0.010 |
| *Erysipelotrichi* | 0.027 | 0.069 | 0.067 | 0.117 |
| *Erysipelotrichales* | 0.027 | 0.069 | 0.067 | 0.117 |
| ***Spirochaetes*** | **2.900** | **3.743** | **2.979** | **3.391** |
| *Spirochaetes* | 2.900 | 3.743 | 2.979 | 3.391 |
| *Spirochaetales* | 2.900 | 3.743 | 2.979 | 3.391 |
| ***Actinobacteria*** | **2.181** | **2.647** | **3.919** | **5.685** |
| *Actinobacteridae* | 1.662 | 1.951 | 3.091 | 4.304 |
| *Actinomycetales* | 1.166 | 1.547 | 2.240 | 3.228 |
| *Nitriliruptorales* | 0.413 | 0.309 | 0.653 | 0.832 |
| *unclassified_Actinobacteridae* | 0.083 | 0.095 | 0.198 | 0.244 |
| *Acidimicrobidae* | 0.295 | 0.322 | 0.362 | 0.624 |
| *Acidimicrobiales* | 0.159 | 0.155 | 0.190 | 0.523 |
| *Acidimicrobidae_*incertae_sedis | 0.102 | 0.112 | 0.134 | 0.076 |
| unclassified*_Acidimicrobidae* | 0.034 | 0.056 | 0.037 | 0.025 |
| unclassified*_Actinobacteria* | 0.144 | 0.163 | 0.261 | 0.335 |
| *Rubrobacteridae* | 0.080 | 0.172 | 0.175 | 0.360 |
| *Solirubrobacterales* | 0.068 | 0.107 | 0.123 | 0.249 |
| unclassified*_Rubrobacteridae* | 0.008 | 0.004 | 0.022 | 0.030 |
| *Rubrobacterales* | 0.004 | 0.056 | 0.030 | 0.076 |
| *Thermoleophilales* | 0.000 | 0.004 | 0.000 | 0.005 |
| *Coriobacteridae* | 0.000 | 0.039 | 0.030 | 0.061 |
| *Coriobacteriales* | 0.000 | 0.039 | 0.030 | 0.061 |
| ***Planctomycetes*** | **1.454** | **1.186** | **2.154** | **0.563** |
| *Planctomycetacia* | 1.454 | 1.186 | 2.154 | 0.563 |
| *Planctomycetales* | 1.454 | 1.186 | 2.154 | 0.563 |
| ***Verrucomicrobia*** | **1.424** | **1.839** | **1.366** | **0.513** |
| *Opitutae* | 0.848 | 1.246 | 0.806 | 0.289 |
| *Puniceicoccales* | 0.617 | 0.898 | 0.593 | 0.152 |
| *Opitutales* | 0.170 | 0.266 | 0.134 | 0.061 |
| *unclassified_Opitutae* | 0.061 | 0.082 | 0.078 | 0.076 |
| *Verrucomicrobiae* | 0.288 | 0.279 | 0.317 | 0.096 |
| *Verrucomicrobiales* | 0.288 | 0.279 | 0.317 | 0.096 |
| unclassified*_"Verrucomicrobia"* | 0.133 | 0.185 | 0.093 | 0.056 |
| *Verrucomicrobia Subdivision3* | 0.087 | 0.052 | 0.056 | 0.010 |
| *Verrucomicrobia Subdivision5* | 0.053 | 0.073 | 0.086 | 0.056 |
| *Spartobacteria* | 0.015 | 0.004 | 0.007 | 0.005 |
| *Spartobacteria* | 0.015 | 0.004 | 0.007 | 0.005 |
| ***Crenarchaeota*** | **0.894** | **1.852** | **2.848** | **0.305** |
| *Thermoprotei* | 0.894 | 1.852 | 2.848 | 0.305 |
| unclassified*_Thermoprotei* | 0.594 | 1.336 | 1.564 | 0.193 |
| *Thermoproteales* | 0.204 | 0.378 | 0.933 | 0.102 |
| *Desulfurococcales* | 0.083 | 0.112 | 0.325 | 0.005 |
| *Acidilobales* | 0.011 | 0.026 | 0.026 | 0.005 |
| ***Cyanobacteria*** | **0.568** | **1.775** | **0.623** | **0.538** |
| *Cyanobacteria* | 0.568 | 1.775 | 0.623 | 0.538 |
| unclassified_*Cyanobacteria* | 0.182 | 0.885 | 0.190 | 0.183 |
| Family IX | 0.159 | 0.584 | 0.067 | 0.061 |
| Family VIII | 0.076 | 0.155 | 0.022 | 0.000 |
| Chloroplast | 0.064 | 0.017 | 0.097 | 0.025 |
| Family I | 0.038 | 0.004 | 0.030 | 0.041 |
| Family XIII | 0.034 | 0.099 | 0.131 | 0.122 |
| Family IV | 0.011 | 0.017 | 0.078 | 0.096 |
| Family V | 0.004 | 0.000 | 0.000 | 0.000 |
| Family X | 0.000 | 0.013 | 0.000 | 0.000 |
| Family XII | 0.000 | 0.000 | 0.007 | 0.005 |
| **WS3** | **0.553** | **0.215** | **0.157** | **0.132** |
| ***Acidobacteria*** | **0.443** | **0.322** | **0.564** | **0.391** |
| *Acidobacteria_*Gp10 | 0.117 | 0.034 | 0.142 | 0.137 |
| *Acidobacteria_*Gp6 | 0.057 | 0.095 | 0.067 | 0.020 |
| *Holophagae* | 0.053 | 0.009 | 0.015 | 0.066 |
| *Holophagales* | 0.042 | 0.009 | 0.015 | 0.066 |
| *Acanthopleuribacterales* | 0.011 | 0.000 | 0.000 | 0.000 |
| *Acidobacteria_*Gp16 | 0.049 | 0.069 | 0.030 | 0.030 |
| *Acidobacteria_*Gp3 | 0.038 | 0.004 | 0.045 | 0.020 |
| *Acidobacteria_*Gp23 | 0.034 | 0.004 | 0.097 | 0.015 |
| *Acidobacteria_*Gp18 | 0.023 | 0.030 | 0.022 | 0.015 |
| *Acidobacteria_*Gp4 | 0.015 | 0.030 | 0.045 | 0.030 |
| *Acidobacteria_*Gp7 | 0.015 | 0.026 | 0.026 | 0.015 |
| unclassified*_"Acidobacteria"* | 0.011 | 0.013 | 0.037 | 0.015 |
| *Acidobacteria_*Gp21 | 0.011 | 0.000 | 0.007 | 0.015 |
| *Acidobacteria_*Gp17 | 0.008 | 0.009 | 0.026 | 0.000 |
| *Acidobacteria_*Gp9 | 0.004 | 0.000 | 0.000 | 0.000 |
| *Acidobacteria_*Gp25 | 0.004 | 0.000 | 0.000 | 0.000 |
| *Acidobacteria_*Gp1 | 0.004 | 0.000 | 0.004 | 0.010 |
| **unclassified*_Archaea*** | **0.386** | **0.696** | **0.638** | **0.188** |
| **OD1** | **0.276** | **0.348** | **0.123** | **0.030** |
| ***Deinococcus-Thermus*** | **0.204** | **0.228** | **0.321** | **0.102** |
| *Deinococci* | 0.204 | 0.228 | 0.321 | 0.102 |
| *Deinococcales* | 0.201 | 0.228 | 0.314 | 0.102 |
| unclassified *Deinococci* | 0.004 | 0.000 | 0.007 | 0.000 |
| **BRC1** | **0.193** | **0.172** | **0.101** | **0.091** |
| ***Tenericutes*** | **0.148** | **0.309** | **0.157** | **0.294** |
| *Mollicutes* | 0.148 | 0.309 | 0.157 | 0.294 |
| *Acholeplasmatales* | 0.133 | 0.296 | 0.142 | 0.264 |
| *Anaeroplasmatales* | 0.008 | 0.000 | 0.000 | 0.015 |
| unclassified*_Mollicutes* | 0.008 | 0.013 | 0.015 | 0.010 |
| **OP11** | **0.148** | **0.159** | **0.105** | **0.041** |
| ***Lentisphaerae*** | **0.125** | **0.185** | **0.134** | **0.051** |
| *Lentisphaeria* | 0.125 | 0.185 | 0.134 | 0.051 |
| *Victivallales* | 0.080 | 0.086 | 0.082 | 0.025 |
| *Lentisphaerales* | 0.027 | 0.030 | 0.026 | 0.010 |
| unclassified*_"Lentisphaeria"* | 0.019 | 0.069 | 0.026 | 0.015 |
| ***Chlamydiae*** | **0.095** | **0.082** | **0.026** | **0.030** |
| *Chlamydiae* | 0.095 | 0.082 | 0.026 | 0.030 |
| *Chlamydiales* | 0.095 | 0.082 | 0.026 | 0.030 |
| ***Chlorobi*** | **0.091** | **0.146** | **0.063** | **0.051** |
| *Chlorobi* | 0.091 | 0.146 | 0.063 | 0.051 |
| *Chlorobiales* | 0.091 | 0.146 | 0.063 | 0.051 |
| ***Fibrobacteres*** | **0.068** | **0.331** | **0.239** | **0.142** |
| *Fibrobacteria* | 0.068 | 0.331 | 0.239 | 0.142 |
| *Fibrobacteriales* | 0.068 | 0.331 | 0.239 | 0.142 |
| ***Deferribacteres*** | **0.053** | **0.021** | **0.060** | **0.096** |
| *Deferribacteres* | 0.053 | 0.021 | 0.060 | 0.096 |
| *Deferribacterales* | 0.053 | 0.021 | 0.060 | 0.096 |
| ***Thermotogae*** | **0.027** | **0.043** | **0.045** | **0.081** |
| *Thermotogae* | 0.027 | 0.043 | 0.045 | 0.081 |
| *Thermotogales* | 0.027 | 0.043 | 0.045 | 0.081 |
| ***Synergistetes*** | **0.023** | **0.047** | **0.063** | **0.041** |
| *Synergistetia* | 0.023 | 0.047 | 0.063 | 0.041 |
| *Synergistetales* | 0.023 | 0.047 | 0.063 | 0.041 |
| ***Fusobacteria*** | **0.019** | **0.116** | **0.097** | **0.299** |
| *Fusobacteria* | 0.019 | 0.116 | 0.097 | 0.299 |
| *Fusobacteriales* | 0.019 | 0.116 | 0.097 | 0.299 |
| ***Gemmatimonadetes*** | **0.019** | **0.073** | **0.075** | **0.005** |
| *Gemmatimonadetes* | 0.019 | 0.073 | 0.075 | 0.005 |
| *Gemmatimonadales* | 0.019 | 0.073 | 0.075 | 0.005 |
| **TM7** | **0.008** | **0.052** | **0.041** | **0.076** |
| **OP10** | **0.004** | **0.009** | **0.000** | **0.000** |
| ***Nitrospira*** | **0.000** | **0.013** | **0.000** | **0.020** |
| *Nitrospira* | 0.000 | 0.013 | 0.000 | 0.020 |
| *Nitrospirales* | 0.000 | 0.013 | 0.000 | 0.020 |
| **SR1** | **0.000** | **0.009** | **0.004** | **0.000** |
