# Supplementary material for: Metatranscriptomic analysis of a high-sulfide aquatic spring reveals insights into sulfur cycling and unexpected aerobic metabolism
Source: PeerJ. 2015 Sep 22;3:e1259. doi: 10.7717/peerj.1259 (PMC4582958; doi:10.7717/peerj.1259)
Supplement: Table S3 — Values shown represent relative abundance values of taxa from metatranscriptomic libraries created from night (22:15), early morning (07:15) and afternoon (12:15) ribodepleted samples. [file peerj-03-1259-s005.docx]

**Supplemental Table 3**. Taxonomic lineages (phylum-, class-, and order-levels) detected from Zodletone mRNA datasets from within metatranscriptomic libraries created from night (22:15), early morning (07:15) and afternoon (12:15) samples.

| Lineage (NCBI Taxonomy):  **Phylum**  Class/Subclass  Order | Relative abundance in each sample (%) | | |
| --- | --- | --- | --- |
|  | 22:15 | 07:30 | 12:15 |
| ***Proteobacteria*** | **13.3687** | **10.0054** | **14.0336** |
| *Deltaproteobacteria* | 3.1061 | 3.6979 | 3.2573 |
| *Desulfuromonadales* | 1.4497 | 1.4855 | 1.2176 |
| Unassigned *Deltaproteobacteria* | 0.5627 | 0.7699 | 0.7463 |
| *Desulfobacterales* | 0.4162 | 0.4608 | 0.4629 |
| *Myxococcales* | 0.3186 | 0.3971 | 0.3956 |
| *Syntrophobacterales* | 0.2210 | 0.3316 | 0.1824 |
| *Desulfovibrionales* | 0.1378 | 0.2417 | 0.2525 |
| *Deltaproteobacteria* MLMS-1 | 0.0000 | 0.0112 | 0.0000 |
| *Gammaproteobacteria* | 3.0429 | 1.8977 | 2.8365 |
| Unassigned *Gammaproteobacteria* | 0.6775 | 0.4796 | 0.8333 |
| *Methylococcales* | 0.5856 | 0.1274 | 0.1515 |
| *Enterobacteriales* | 0.4880 | 0.3578 | 0.5751 |
| *Chromatiales* | 0.2698 | 0.1723 | 0.2834 |
| *Legionellales* | 0.2383 | 0.1368 | 0.1824 |
| *Vibrionales* | 0.1923 | 0.1068 | 0.1319 |
| *Alteromonadales* | 0.1636 | 0.1649 | 0.1319 |
| *Oceanospirillales* | 0.1206 | 0.0824 | 0.1515 |
| *Thiotrichales* | 0.0919 | 0.0862 | 0.1431 |
| *Pasteurellales* | 0.0861 | 0.0393 | 0.0561 |
| *Pseudomonadales* | 0.0632 | 0.0899 | 0.0842 |
| S oxidizing symbionts | 0.0459 | 0.0262 | 0.0449 |
| *Xanthomonadales* | 0.0201 | 0.0281 | 0.0673 |
| *Betaproteobacteria* | 2.2851 | 0.8936 | 2.2192 |
| *Unassigned Betaproteobacteria* | 1.7999 | 0.5564 | 1.3214 |
| *Burkholderiales* | 0.2957 | 0.2079 | 0.3339 |
| Uncultured betaproteobacterium CBNPD1_BACclone578 | 0.1321 | 0.0150 | 0.5275 |
| *Neisseriales* | 0.0201 | 0.0000 | 0.0000 |
| *Nitrosomonadales* | 0.0201 | 0.0131 | 0.0000 |
| *Rhodocyclales* | 0.0172 | 0.0169 | 0.0365 |
| *Alphaproteobacteria* | 2.2793 | 1.6129 | 2.8140 |
| *Rhodospirillales* | 0.8813 | 0.4515 | 0.8080 |
| *Unassigned Alphaproteobacteria* | 0.4679 | 0.2754 | 0.4854 |
| *Rhodobacterales* | 0.4335 | 0.4252 | 0.7098 |
| *Rhizobiales* | 0.4220 | 0.3784 | 0.4545 |
| *Sphingomonadales* | 0.0287 | 0.0487 | 0.0365 |
| *Caulobacterales* | 0.0258 | 0.0244 | 0.0196 |
| alphaproteobacterium_BAL199 | 0.0201 | 0.0000 | 0.0196 |
| Unassigned *Proteobacteria* | 1.9722 | 1.5680 | 2.1435 |
| *Epsilonproteobacteria* | 0.6631 | 0.4740 | 0.7323 |
| *Campylobacterales* | 0.4392 | 0.3054 | 0.4236 |
| Unassigned *Epsilonproteobacteria* | 0.1780 | 0.0955 | 0.1459 |
| Unclassified *Epsilonproteobacteria*, *Sulfurovum* Group | 0.0459 | 0.0731 | 0.1627 |
| Unassigned *Delta/Epsilonproteobacteria* | 0.0201 | 0.0206 | 0.0309 |
| *Zetaproteobacteria* | 0.000 | 0.0094 | 0.0000 |
| *Mariprofundales* | 0.000 | 0.0094 | 0.0000 |
| **Unassigned *Bacteria*** | **7.9058** | **8.9545** | **10.3779** |
| ***Bacteroidetes*** | **3.2008** | **4.2150** | **3.5154** |
| *Bacteroidia* | 1.4784 | 1.7909 | 1.9471 |
| *Bacteroidales* | 1.4784 | 1.7909 | 1.9471 |
| Unassigned *Bacteroidetes* | 0.9129 | 1.2757 | 0.7828 |
| *Flavobacteria* | 0.3703 | 0.5864 | 0.3900 |
| *Flavobacteriales* | 0.3186 | 0.5208 | 0.3058 |
| Unassigned *Flavobacteria* | 0.0517 | 0.0656 | 0.0842 |
| *Sphingobacteria* | 0.2268 | 0.2529 | 0.2104 |
| *Sphingobacteriales* | 0.2268 | 0.2529 | 0.2104 |
| *Cytophagia* | 0.2124 | 0.2979 | 0.1852 |
| *Cytophagales* | 0.2124 | 0.2979 | 0.1852 |
| *Bacteroidetes* environmental metagenome | 0.0172 | 0.0000 | 0.0000 |
| ***Firmicutes*** | **3.1290** | **3.8066** | **4.0204** |
| *Clostridia* | 1.9779 | 2.3285 | 2.4914 |
| *Clostridiales* | 1.5530 | 1.8265 | 1.9106 |
| Unassigned *Clostridia* | 0.2038 | 0.2510 | 0.2834 |
| *Thermoanaerobacterales* | 0.1837 | 0.2192 | 0.2581 |
| *Halanaerobiales* | 0.0373 | 0.0318 | 0.0393 |
| Unassigned *Firmicutes* | 0.5770 | 0.7793 | 0.7014 |
| *Bacilli* | 0.4938 | 0.6351 | 0.7238 |
| *Bacillales* | 0.3588 | 0.4702 | 0.5639 |
| *Lactobacillales* | 0.0976 | 0.1311 | 0.1234 |
| Unassigned *Bacilli* | 0.0373 | 0.0337 | 0.0365 |
| *Erysipelotrichi* | 0.0517 | 0.0393 | 0.0842 |
| *Erysipelotrichales* | 0.0517 | 0.0393 | 0.0842 |
| *Negativicutes* | 0.0287 | 0.0244 | 0.0196 |
| *Selenomonadales* | 0.0287 | 0.0244 | 0.0196 |
| ***Euryarchaeota*** | **2.9023** | **3.7523** | **3.2180** |
| *Methanomicrobia* | 1.7827 | 2.4634 | 1.4084 |
| *Methanosarcinales* | 1.1397 | 1.6223 | 0.9707 |
| *Methanomicrobiales* | 0.5770 | 0.7550 | 0.3731 |
| Unassigned *Methanomicrobia* | 0.0660 | 0.0862 | 0.0645 |
| *Thermococci* | 0.3502 | 0.3859 | 0.4545 |
| *Thermococcales* | 0.3502 | 0.3859 | 0.4545 |
| Unassigned *Euryarchaeota* | 0.2842 | 0.3653 | 0.3058 |
| *Halobacteria* | 0.2268 | 0.1911 | 0.4966 |
| *Halobacteriales* | 0.2268 | 0.1911 | 0.4966 |
| *Thermoplasmata* | 0.1636 | 0.2398 | 0.4770 |
| *Candidatus Aciduliprofundum* | 0.1636 | 0.2398 | 0.4770 |
| *Methanococci* | 0.0373 | 0.0337 | 0.0505 |
| *Methanococcales* | 0.0373 | 0.0337 | 0.0505 |
| *Archaeoglobi* | 0.0258 | 0.0281 | 0.0253 |
| *Archaeoglobales* | 0.0258 | 0.0281 | 0.0253 |
| *Methanobacteria* | 0.0000 | 0.0187 | 0.0000 |
| *Methanobacteriales* | 0.0000 | 0.0187 | 0.0000 |
| Uncultured_methanogenic_archaeon_RC-1 | 0.0000 | 0.0169 | 0.0000 |
| **Unassigned cellular organisms** | **2.1702** | **1.2514** | **2.9739** |
| ***Actinobacteria*** | **1.2114** | **1.1071** | **2.7691** |
| *Actinobacteridae* | 1.0909 | 0.9629 | 2.4858 |
| *Actinomycetales* | 1.0708 | 0.9423 | 2.4465 |
| *Bifidobacteriales* | 0.0172 | 0.0169 | 0.0253 |
| *Unassigned Actinobacteridae* | 0.0029 | 0.0037 | 0.0140 |
| *Rubrobacteridae* | 0.0545 | 0.0506 | 0.1150 |
| *Rubrobacterales* | 0.0459 | 0.0356 | 0.0673 |
| *Solirubrobacterales* | 0.0000 | 0.0150 | 0.0477 |
| Unassigned *Actinobacteria* | 0.0373 | 0.0637 | 0.1010 |
| *Acidimicrobidae* | 0.0144 | 0.0000 | 0.0000 |
| *Acidimicrobiales* | 0.0144 | 0.0000 | 0.0000 |
| *Coriobacteridae* | 0.0144 | 0.0300 | 0.0673 |
| *Coriobacteriales* | 0.0144 | 0.0300 | 0.0673 |
| ***Spirochaetes*** | **1.0679** | **1.5118** | **1.1559** |
| *Spirochaetes* | 1.0679 | 1.5118 | 1.1559 |
| *Spirochaetales* | 1.0679 | 1.5118 | 1.1559 |
| ***Chloroflexi*** | **0.8583** | **1.0041** | **1.0998** |
| *Chloroflexi* | 0.5598 | 0.5957 | 0.6369 |
| *Chloroflexales* | 0.3703 | 0.4140 | 0.4208 |
| Unassigned *Chloroflexi* | 0.0976 | 0.0937 | 0.1206 |
| *Herpetosiphonales* | 0.0919 | 0.0880 | 0.0954 |
| *Thermomicrobia* | 0.1407 | 0.1349 | 0.2160 |
| *Sphaerobacterales* | 0.0574 | 0.0656 | 0.0954 |
| *Thermomicrobiales* | 0.0459 | 0.0450 | 0.0505 |
| Unassigned *Thermomicrobia* | 0.0373 | 0.0244 | 0.0701 |
| Unassigned *Chloroflexi* | 0.1120 | 0.1293 | 0.1655 |
| *Dehalococcoidetes* | 0.0459 | 0.1274 | 0.0589 |
| *Dehalococcoides* | 0.0459 | 0.1274 | 0.0589 |
| Unclassified *Chloroflexi* bacterium | 0.0000 | 0.0169 | 0.0224 |
| ***Cyanobacteria*** | **0.7004** | **1.2607** | **0.3844** |
| *Cyanobacteria* | 0.7004 | 1.2607 | 0.3844 |
| *Chroococcales* | 0.3186 | 0.5601 | 0.1094 |
| Unassigned *Cyanobacteria* | 0.3014 | 0.5526 | 0.1964 |
| *Nostocales* | 0.0459 | 0.0824 | 0.0168 |
| *Oscillatoriales* | 0.0344 | 0.0562 | 0.0196 |
| *Acaryochloris* | 0.0000 | 0.0094 | 0.0000 |
| *Gleobacterales* | 0.0000 | 0.0000 | 0.0140 |
| *Prochlorales* | 0.0000 | 0.0000 | 0.0281 |
| ***Deinococcus-Thermus*** | **0.4249** | **0.3822** | **0.8417** |
| *Deinococci* | 0.4249 | 0.3822 | 0.8417 |
| *Deinococcales* | 0.3818 | 0.3503 | 0.7547 |
| *Thermales* | 0.0373 | 0.0244 | 0.0814 |
| ***Planctomycetes*** | **0.3445** | **0.3016** | **0.4208** |
| *Planctomycetacia* | 0.3445 | 0.3016 | 0.4208 |
| *Planctomycetales* | 0.3445 | 0.3016 | 0.4208 |
| ***Crenarchaeota*** | **0.2842** | **0.3915** | **0.3507** |
| *Thermoprotei* | 0.2785 | 0.3915 | 0.3451 |
| *Sulfolobales* | 0.2526 | 0.3128 | 0.2806 |
| Unassigned *Thermoprotei* | 0.0258 | 0.0187 | 0.0224 |
| *Desulfurococcales* | 0.0000 | 0.0150 | 0.0000 |
| *Thermoproteales* | 0.0000 | 0.0450 | 0.0421 |
| Unassigned *Crenarchaeota* | 0.0057 | 0.0000 | 0.0056 |
| ***Verrucomicrobia*** | **0.2239** | **0.2248** | **0.2048** |
| *Verrucomicrobiae* | 0.1263 | 0.0974 | 0.0561 |
| *Verrucomicrobiales* | 0.1263 | 0.0974 | 0.0561 |
| Unassigned *Verrucomicrobia* | 0.0402 | 0.0393 | 0.0758 |
| *Opitutae* | 0.0344 | 0.0674 | 0.0561 |
| *Opitutales* | 0.0344 | 0.0674 | 0.0561 |
| *Spartobacteria* | 0.0230 | 0.0206 | 0.0168 |
| *Chthoniobacter* | 0.0230 | 0.0206 | 0.0168 |
| ***Thermotogae*** | **0.1981** | **0.2192** | **0.1992** |
| *Thermotogae* | 0.1981 | 0.2192 | 0.1992 |
| *Thermotogales* | 0.1981 | 0.2192 | 0.1992 |
| ***Chlorobi*** | **0.1608** | **0.1911** | **0.1908** |
| *Chlorobia* | 0.1608 | 0.1911 | 0.1908 |
| *Chlorobiales* | 0.1608 | 0.1911 | 0.1908 |
| **Unassigned *Archaea*** | **0.1435** | **0.1967** | **0.0056** |
| ***Synergistetes*** | **0.0861** | **0.0824** | **0.1178** |
| *Synergistia* | 0.0861 | 0.0824 | 0.1178 |
| *Synergistales* | 0.0861 | 0.0824 | 0.1178 |
| **Candidate Division WWE1** | **0.0832** | **0.1480** | **0.1066** |
| Candidatus *Cloacomonas* | 0.0832 | 0.1480 | 0.1066 |
| ***Aquificae*** | **0.0804** | **0.1105** | **0.1291** |
| *Aquificae* | 0.0804 | 0.1105 | 0.1291 |
| *Aquificales* | 0.0804 | 0.1105 | 0.1291 |
| ***Thermobaculum terrenum*** | **0.0632** | **0.0656** | **0.1150** |
| ***Lentisphaerae*** | **0.0545** | **0.0487** | **0.0898** |
| *Lentisphaerae* | 0.0545 | 0.0487 | 0.0898 |
| *Lentisphaerales* | 0.0287 | 0.0112 | 0.0589 |
| *Victivallales* | 0.0258 | 0.0337 | 0.0281 |
| ***Bacteria* environmental metagenomes** | **0.0517** | **0.0749** | **0.0477** |
| ***Archaea* environmental metagenomes** | **0.0517** | **0.0712** | **0.0477** |
| **Unassigned *Bacteroidetes/Chlorobi group*** | **0.0431** | **0.0749** | **0.0449** |
| ***Tenericutes*** | **0.0402** | **0.0412** | **0.0337** |
| *Mollicutes* | 0.0402 | 0.0412 | 0.0337 |
| *Acholeplasmatales* | 0.0287 | 0.0393 | 0.0224 |
| ***Gemmatimonadetes*** | **0.0402** | **0.0262** | **0.0337** |
| *Gemmatimonadetes* | 0.0402 | 0.0262 | 0.0337 |
| *Gemmatimonadales* | 0.0402 | 0.0262 | 0.0337 |
| ***Acidobacteria*** | **0.0373** | **0.0506** | **0.0168** |
| *Candidatus Koribacter* | 0.0287 | 0.0262 | 0.0000 |
| *Unassigned Acidobacteria* | 0.0086 | 0.0112 | 0.0168 |
| *Acidobacteria* | 0.0000 | 0.0131 | 0.0000 |
| *Acidobacteriales* | 0.0000 | 0.0131 | 0.0000 |
| ***Fusobacteria*** | **0.0287** | **0.0431** | **0.0309** |
| *Fusobacteria* | 0.0287 | 0.0431 | 0.0309 |
| *Fusobacteriales* | 0.0287 | 0.0431 | 0.0309 |
| ***Fibrobacteres*** | **0.0258** | **0.0525** | **0.0168** |
| *Fibrobacteres* | 0.0258 | 0.0525 | 0.0168 |
| *Fibrobacteriales* | 0.0258 | 0.0525 | 0.0168 |
| **Unassigned *Chlamydiae/Verrucomicrobia* group** | **0.0258** | **0.0244** | **0.0309** |
| ***Thaumarchaeota*** | **0.0230** | **0.0225** | **0.0168** |
| *Thaumarchaeota* | 0.0230 | 0.0225 | 0.0168 |
| Unassigned *Thaumarchaeota* | 0.0230 | 0.0075 | 0.0168 |
| *Cenarchaeales* | 0.0000 | 0.0150 | 0.0000 |
| **Candidate Division TM7** | **0.0172** | **0.0000** | **0.0140** |
| **Candidatus *Poribacteria*** | **0.0000** | **0.0112** | **0.0000** |
| ***Deferribacteres*** | **0.0000** | **0.0225** | **0.0000** |
| *Deferribacteres* | 0.0000 | 0.0225 | 0.0000 |
| *Deferribacterales* | 0.0000 | 0.0225 | 0.0000 |
| ***Korarcheaota*** | **0.0000** | **0.0094** | **0.0000** |
| *Korarchaeota* | 0.0000 | 0.0094 | 0.0000 |
| *Korarchaeota* | 0.0000 | 0.0094 | 0.0000 |
| ***Nitrospirae*** | **0.0000** | **0.0000** | **0.0168** |
| *Nitrospira* | 0.0000 | 0.0000 | 0.0168 |
| *Nitrospirales* | 0.0000 | 0.0000 | 0.0168 |
